# Charge–Capacitance Channel Decomposition Reveals Fabrication-Tolerant Design Windows for Disk Triboelectric Nanogenerators

**DOI:** 10.3390/ma19122607

**Published:** 2026-06-17

**Authors:** Shenchen Liu, Yangshi Shao, Xuhong Feng, Zehui Lin, Xiaoming Jing, Everett X. Wang

**Affiliations:** 1 Beijing Institute of Nanoenergy and Nanosystems, Chinese Academy of Sciences, Beijing 101400, China; liushenchen@binn.cas.cn (S.L.); shaoyangshi@binn.cas.cn (Y.S.); fengxuhong@binn.cas.cn (X.F.); linzehui@binn.cas.cn (Z.L.); jingxiaoming@binn.cas.cn (X.J.); 2 School of Nanoscience and Engineering, University of Chinese Academy of Sciences, Beijing 100049, China

**Keywords:** triboelectric nanogenerator, disk TENG, transformer, surrogate modeling, mechanism landscape, fabrication-tolerant design windows, geometric robustness

## Abstract

Disk triboelectric nanogenerator (TENG) design pursues high structural figure of merit ( ${\mathrm{FOM}}_{S}$ ), yet nominal peak designs often sit in regions with steep geometric gradients; under a controlled ±10% symmetric perturbation proxy, worst-case ${\mathrm{FOM}}_{S}$ retention near the peak frontier falls to 2.7%. We decompose ${\mathrm{FOM}}_{S}$ into a charge-transfer channel ( $Q_{\mathrm{sc},\mathrm{MACRS}}$ ) and a capacitance channel ( $C_{\mathrm{sum}}^{-1}$ ), and train a multi-output surrogate with a physics consistency constraint on 1944 COMSOL simulations to jointly predict $Q_{\mathrm{sc},\mathrm{MACRS}}$ , $C_{\mathrm{sum}}^{-1}$ , and ${\mathrm{FOM}}_{S}$ across electrode-pair number, dielectric-thickness-to-radius ratio ( h/R ), air-gap-to-radius ratio ( d/R ), and dielectric constant. Evaluating 7776 design points reveals that 58.6% of the explored space is charge-dominant, 36.1% mixed, and 5.3% capacitance-dominant; raising dielectric constant shifts the mechanism toward capacitance-limited behavior, while a larger air gap reinforces charge-limited behavior. Mixed-regime windows tolerate the same perturbation proxy far better than peak- ${\mathrm{FOM}}_{S}$ candidates, supplying candidate design windows for pre-fabrication screening within the validated simulation domain. The surrogate reaches pooled out-of-distribution ${\mathrm{FOM}}_{S}$ $R_{\log_{10}}^{2}=0.914$ on 43 unseen structural and dielectric combinations. Delivered through an open-source Streamlit interface, the channel decomposition, mechanism mapping, and tolerance screening let designers identify the limiting mechanism and select candidate designs that are expected to tolerate geometric variation within the validated simulation domain, prior to fabrication.

## 1. Introduction

Triboelectric nanogenerator (TENG) converts distributed mechanical energy into electricity through contact electrification and electrostatic induction [1,2,3,4,5,6,7]. Among the various TENG configurations, the disk geometry is particularly well suited to rotational and cyclic excitation [8,9,10,11,12]. Its output depends on four interacting parameters: electrode-pair number, dielectric-thickness-to-radius ratio ( h/R ), air-gap-to-radius ratio ( d/R ), and dielectric constant. These parameters interact nonlinearly, so exhaustive finite-element sweeps are costly and rarely produce design guidance that generalizes [3,13,14].

Most disk-TENG optimization studies pursue ${\mathrm{FOM}}_{S}$ maxima as if the underlying design were dimensionally fixed. In practice, air gaps and dielectric thicknesses are subject to fabrication variability, and the tolerance band tightens precisely where the nominal ${\mathrm{FOM}}_{S}$ peak sits, because the gradient of the figure of merit is steepest in the same regions that produce the highest output. Under a controlled ±10% geometric perturbation proxy, a design that wins on paper can therefore lose much of its predicted output once geometric variation is folded back in. A surrogate trained on ${\mathrm{FOM}}_{S}$ alone cannot tell whether this gap comes from charge transfer, capacitance, or both. Without that distinction, a designer has no way to judge where a nominal optimum is worth pursuing and where it is too fragile to build.

Surrogate models and interpretable machine learning have begun to ease this bottleneck [15,16,17,18,19]. Han et al. [20] showed that a surrogate trained on structural inputs can predict disk-TENG performance and that SHAP-based feature importance [21,22,23] can reveal complex variable interactions. Three questions remain open for translating such models into practice: where in the design space a given physical pathway becomes the local bottleneck, which adjustments stay effective inside that regime, and whether a high- ${\mathrm{FOM}}_{S}$ optimum tolerates geometric variation at fabrication scales [24,25].

This gap originates in the definition of ${\mathrm{FOM}}_{S}$ itself. ${\mathrm{FOM}}_{S}$ factors into a charge-transfer contribution ( $Q_{\mathrm{sc},\mathrm{MACRS}}^{2}$ ) and a capacitance-related contribution ( $C_{\mathrm{sum}}^{-1}$ ), so two designs with identical ${\mathrm{FOM}}_{S}$ values can be limited by entirely different mechanisms [26,27,28,29,30,31,32,33,34]. A surrogate that predicts ${\mathrm{FOM}}_{S}$ as a single scalar cannot distinguish whether a design is charge-limited, capacitance-limited, or balanced. When a structural variable improves the charge pathway in one region but mainly shifts the capacitance pathway in another, a single global importance score marks it as relevant everywhere while giving no local guidance on how to use it.

We train a multi-output surrogate with a physics consistency constraint on 1944 COMSOL Multiphysics 6.4 simulations to jointly predict $Q_{\mathrm{sc},\mathrm{MACRS}}$ , $C_{\mathrm{sum}}^{-1}$ , and ${\mathrm{FOM}}_{S}$ across the four-dimensional parameter space. Charge-dominant, capacitance-dominant, and mixed regimes coexist, each calling for a different tuning strategy. The peak- ${\mathrm{FOM}}_{S}$ frontier sits in a region of high perturbation sensitivity, whereas mixed-regime windows offer a more robust design target. We expose the same numerical core through an open Streamlit tool, so candidates that tolerate fabrication variation can be screened within the validated domain before any device is built.

## 2. Materials and Methods

The same numerical core supports every figure in this paper and is exposed through an open Streamlit interface. Users enter four design variables (*n*, h/R , d/R , ε ) and the interface returns a ${\mathrm{FOM}}_{S}$ prediction, the local mechanism label, and a tolerance score, so candidates can be screened before COMSOL or experimental work begins. Implementation details are in Section 2.5 and in the Supporting Information.

### 2.1. ${\mathrm{FOM}}_{S}$ Definition and Channel Decomposition

${\mathrm{FOM}}_{S}$ cannot be treated as a single scalar target, because it factors into two physically interpretable contributions: a charge-transfer term ( $Q_{\mathrm{sc},\mathrm{MACRS}}^{2}$ ) and a capacitance-related term ( $C_{\mathrm{sum}}^{-1}$ ). Following the simulation formulation [26,27,35], the physically reconstructed quantity is

(1) $${\mathrm{FOM}}_{S,\mathrm{phys}}=\frac{2n\varepsilon_{0}}{\sigma^{2}\pi^{2}R^{3}}\;Q_{\mathrm{sc},\mathrm{MACRS}}^{2}\;C_{\mathrm{sum}}^{-1},$$

where $Q_{\mathrm{sc},\mathrm{MACRS}}$ is the short-circuit charge difference under the MACRS condition, $C_{\mathrm{sum}}^{-1}=C_{\mathrm{start}}^{-1}+C_{\mathrm{end}}^{-1}$ is the sum of inverse capacitances at the start and end states, *n* is the electrode-pair number, $\varepsilon_{0}$ is the vacuum permittivity, σ is the surface charge density, and *R* is the disk radius. This factorization reveals three independent routes through which ${\mathrm{FOM}}_{S}$ can increase: stronger charge transfer alone, a more favorable capacitance pathway alone, or simultaneous improvement of both.

Predicting ${\mathrm{FOM}}_{S}$ as a single output provides no way to distinguish whether two similar-performing designs are limited by the same mechanism. The surrogate was therefore trained to predict three outputs simultaneously: $Q_{\mathrm{sc},\mathrm{MACRS}}$ , $C_{\mathrm{sum}}^{-1}$ , and a direct ${\mathrm{FOM}}_{S}$ head ( ${\mathrm{FOM}}_{S,\mathrm{direct}}$ ). A physically reconstructed ${\mathrm{FOM}}_{S,\mathrm{phys}}$ —recomputed from the predicted channel outputs via Equation (1)—served as an internal consistency reference throughout training.

**Figure 1:**
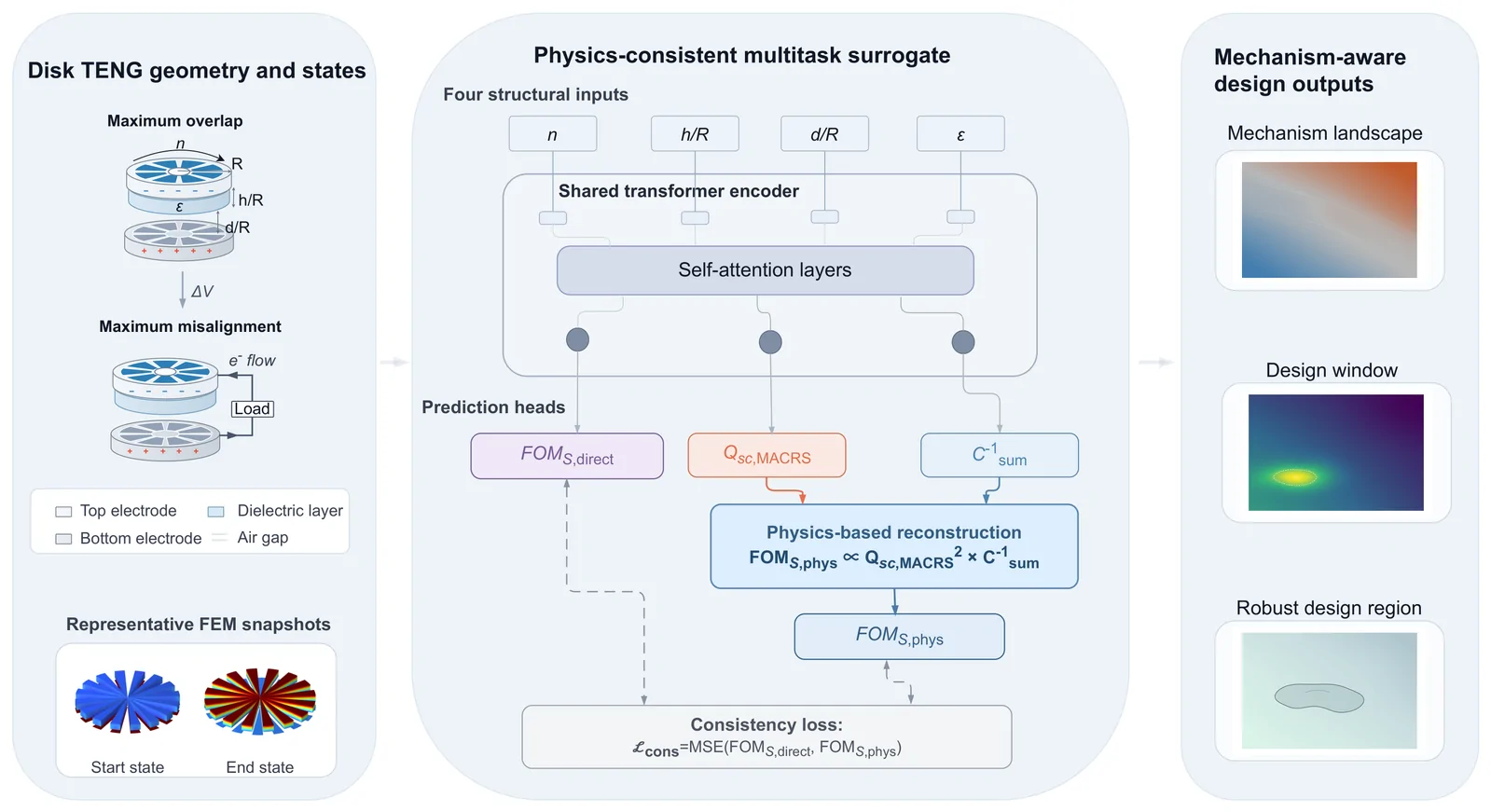
Workflow overview from disk-TENG geometry and representative electrostatic states to the shared transformer encoder, multitask prediction heads, physics-based reconstruction branch, consistency constraint, and mechanism classification outputs.

Figure 1 summarizes this workflow.

### 2.2. Data Generation, Preprocessing, and Evaluation Protocol

All models were trained on the final processed training dataset disk_teng_training_processed.csv, generated from the COMSOL simulation campaign and containing 1944 valid disk-TENG designs sampled on a structured four-dimensional grid. The primary design variables were electrode-pair number n∈{2,4,8,16,32,64} and dielectric constant ε∈{1,2,3,5,7,10} . The two geometric ratios were

$$\begin{array}{cc} d/R & \in \{0.03125,0.0625,0.125,0.25,0.5,1.0\},\\ h/R & \in \{0.00390625,0.0078125,0.015625,0.03125,0.0625,0.125,0.25,0.5,1.0\}. \end{array}$$

After loading, $C_{\mathrm{sum}}^{-1}$ was constructed as $C_{\mathrm{start}}^{-1}+C_{\mathrm{end}}^{-1}$ , and entries with non-positive $Q_{\mathrm{sc},\mathrm{MACRS}}$ , $C_{\mathrm{start}}^{-1}$ , $C_{\mathrm{end}}^{-1}$ , $C_{\mathrm{sum}}^{-1}$ , or ${\mathrm{FOM}}_{S}$ were excluded.

The three targets span markedly different dynamic ranges— $Q_{\mathrm{sc},\mathrm{MACRS}}$ covers approximately 6.7 orders of magnitude, $C_{\mathrm{sum}}^{-1}$ about 2.5 orders, and ${\mathrm{FOM}}_{S}$ about 11.3 orders—so all three were transformed to $\log_{10}$ space before scaling. Distribution statistics in $\log_{10}$ space—including range, median, interquartile range, and skewness—are reported in Table S3, and Kolmogorov–Smirnov tests confirming partition homogeneity are reported in Table S4. Each target was normalized independently with a MinMax transform fitted only on the training partition, while the four input variables were scaled to [0, 1]. The 1944 samples were split 80/10/10 into training, validation, and test partitions under a fixed seed.

The evaluation protocol has three levels, each addressing a different question. The random held-out test split (195 points) probes interpolation among neighboring conditions on the training grid. Five-fold cross-validation confirms that this result reflects stable interpolation rather than a lucky split. Three external validation sets (V1, V2, V3, totaling 43 unseen structural and dielectric combinations) assess out-of-distribution (OOD) generalization: V1 isolates extrapolation to unseen *n* values, V2 applies simultaneous shifts in all four variables, and V3 spans three scenarios with both seen and unseen *n*. Per-set parameter listings are tabulated in the Supporting Information. This hierarchy separates extrapolation to unseen *n* values from more demanding shifts in geometry and material. The model is validated within the sampled parameter domain. Extending it to other TENG types (e.g., planar, cylindrical) or parameter ranges outside the training grid would require new simulations and retraining—a characteristic of all surrogate models, not specific to this work.

Regression performance was reported after inverse transformation using mean absolute error, root-mean-square error, coefficient of determination, and mean absolute percentage error. Because ${\mathrm{FOM}}_{S}$ spans more than ten orders of magnitude, scale-invariant metrics were also computed in $\log_{10}$ space, especially $R_{\log_{10}}^{2}$ and ${\mathrm{MAE}}_{\log_{10}}$ . Internal agreement between ${\mathrm{FOM}}_{S,\mathrm{direct}}$ and ${\mathrm{FOM}}_{S,\mathrm{phys}}$ was quantified through mean absolute error, Pearson correlation, and Spearman correlation.

### 2.3. Physics-Constrained Multi-Output Surrogate Model

The final model uses a shared encoder to produce all three predictions from the same internal representation, keeping the learned features consistent across channels [36,37,38,39,40]. Each of the four scalar design variables is projected into a latent vector, forming a sequence of four tokens. A transformer encoder processes this sequence without positional encoding, so the attention layers infer variable relationships from the input values rather than from their order. The shared representation is then flattened and passed to three regression heads that predict normalized $\log_{10}\left(Q_{\mathrm{sc},\mathrm{MACRS}}\right)$ , $\log_{10}\left(C_{\mathrm{sum}}^{-1}\right)$ , and $\log_{10}\left({\mathrm{FOM}}_{S,\mathrm{direct}}\right)$ independently. All hyperparameters—embedding dimension, number of attention heads, encoder layers, dropout, optimizer, and learning rate schedule—are listed in Table S1 [41,42,43]. The same seed and data split were reused across all downstream analyses, tying every reported figure to one fixed model instance.

Training minimized three task losses and one consistency loss. Let $L_{q}$ , $L_{c}$ , and $L_{f}$ denote the mean-squared errors for normalized $\log_{10}\left(Q_{\mathrm{sc},\mathrm{MACRS}}\right)$ , $\log_{10}\left(C_{\mathrm{sum}}^{-1}\right)$ , and $\log_{10}\left({\mathrm{FOM}}_{S,\mathrm{direct}}\right)$ . A reconstructed ${\mathrm{FOM}}_{S,\mathrm{phys}}$ was computed from the predicted channels in $\log_{10}$ space and mapped into the same normalized range as ${\mathrm{FOM}}_{S,\mathrm{direct}}$ . The total objective was

(2) $$L_{\mathrm{total}}=\lambda_{q}L_{q}+\lambda_{c}L_{c}+\lambda_{f}L_{f}+\lambda_{\mathrm{cons}}L_{\mathrm{cons}}$$

with $\lambda_{q}=\lambda_{c}=\lambda_{f}=1.0$ and $\lambda_{\mathrm{cons}}=0.3$ . The consistency term keeps the direct ${\mathrm{FOM}}_{S}$ head aligned with the channel decomposition that later supports the mechanism maps and robustness analysis.

### 2.4. Mechanism Landscape and Design-Space Construction

The first analysis constructed a dominance landscape in mechanism space. All simulated designs were projected onto the $\left(\log Q,\log C^{-1}\right)$ plane, where $\log Q=\log_{10}\left(Q_{\mathrm{sc},\mathrm{MACRS}}^{2}\right)$ represents the charge-transfer contribution and $\log C^{-1}=\log_{10}\left(C_{\mathrm{sum}}^{-1}\right)$ represents the capacitance contribution. A *k*-nearest-neighbor density mask [44] filtered out regions with too few nearby data points. In each remaining cell, a local variance ratio determined which channel drove more of the ${\mathrm{FOM}}_{S}$ variation. The local charge fraction

(3) $$f_{\mathrm{charge}}=\frac{\mathrm{std}(\log Q)}{\mathrm{std}\left(\log Q\right)+\mathrm{std}\left(\log C^{-1}\right)}$$

was computed over the 50 nearest neighbors at each grid location. This fraction ranges from 0 (fully capacitance-driven) to 1 (fully charge-driven) and reflects how much of the local ${\mathrm{FOM}}_{S}$ variation is attributable to the charge channel. Under the reference setting, cells were classified as charge-dominant when $f_{\mathrm{charge}}>0.62$ , capacitance-dominant when $f_{\mathrm{charge}}<0.38$ , and mixed otherwise. The threshold of 0.62 was chosen to impose a moderately asymmetric partition rather than an equal 50/50 split; the sensitivity analysis in Figure S6 confirms that the qualitative conclusions remain robust across the range 0.55–0.70. All area fractions reported for the mechanism-space dominance map correspond to this reference setting and should be read as setting-specific summaries rather than invariant physical constants.

The second analysis mapped these mechanism labels back into the structural design space using local sensitivity. Dense predictions were generated on a grid containing six *n* values, six ε values, 12 geometrically spaced d/R values (0.03125 to 1.0), and 18 geometrically spaced h/R values (0.00390625 to 1.0), yielding 7776 valid design points. At each point, mechanism dominance was determined from finite-difference sensitivities in log space [45,46]. With a multiplicative step of $\delta_{\log}=0.02$ , local gradients of $\log Q_{\mathrm{sc},\mathrm{MACRS}}$ and $\log C_{\mathrm{sum}}^{-1}$ with respect to logn and log(h/R) were estimated, and the corresponding channel sensitivities were defined as

(4) $$\begin{array}{cc} S_{\mathrm{charge}} & =\sqrt{{\left(2\frac{\partial \log Q_{\mathrm{sc},\mathrm{MACRS}}}{\partial \log n}\right)}^{2}+{\left(2\frac{\partial \log Q_{\mathrm{sc},\mathrm{MACRS}}}{\partial \log(h/R)}\right)}^{2}} \end{array}$$

(5) $$\begin{array}{cc} S_{\mathrm{cap}} & =\sqrt{{\left(\frac{\partial \log C_{\mathrm{sum}}^{-1}}{\partial \log n}\right)}^{2}+{\left(\frac{\partial \log C_{\mathrm{sum}}^{-1}}{\partial \log(h/R)}\right)}^{2}} \end{array}$$

The design-space fraction $f_{\mathrm{charge}}=S_{\mathrm{charge}}/\left(S_{\mathrm{charge}}+S_{\mathrm{cap}}\right)$ was classified with the same 0.62 threshold. The mechanism-space map and the design-space map answer different questions. The former shows which channel drives more of the observed ${\mathrm{FOM}}_{S}$ variation across designs; the latter shows which channel responds more to a small parameter change at a given operating point.

To ensure that qualitative conclusions were not artifacts of a single parameter choice, the neighborhood size was varied from 20 to 100 and the dominance threshold from 0.55 to 0.70 in the mechanism-space analysis. In the design-space analysis, the h/R sampling density was varied from 9 to 36 points.

### 2.5. Geometric Tolerance Proxy and Open Design Interface

Geometric robustness was approximated by applying symmetric perturbations to d/R and h/R , the two variables most sensitive to fabrication variation. For each point in the representative (n,h/R) maps at fixed ε and d/R , four corner perturbations were generated by applying ±10% changes to both d/R and h/R , yielding five ${\mathrm{FOM}}_{S}$ values per design point. Robustness was summarized by the coefficient of variation,

(6) $$\mathrm{CV}=\frac{\mathrm{std}({\mathrm{FOM}}_{S})}{\mathrm{mean}({\mathrm{FOM}}_{S})}$$

and by the worst-case retention ratio, defined as the minimum perturbed ${\mathrm{FOM}}_{S}$ divided by the baseline value. Regions with low CV and high retention were treated as candidate windows with low perturbation sensitivity [47,48].

The open Streamlit interface uses the same surrogate, mechanism metrics, and robustness screening as the main-text figures. Given the four design variables, it returns the predicted ${\mathrm{FOM}}_{S}$ , the local mechanism label, and a tolerance score from the same perturbation protocol, so individual designs and broader explorations follow the same criteria as the figures in this paper. The full implementation and associated resources are provided through the study repository described in the Data Availability Statement.

## 3. Results

### 3.1. Model Framework and Predictive Capability

The held-out test split yielded $R_{\log_{10}}^{2}$ values of 0.9898 for $Q_{\mathrm{sc},\mathrm{MACRS}}$ , 0.9966 for $C_{\mathrm{sum}}^{-1}$ , and 0.9817 for ${\mathrm{FOM}}_{S,\mathrm{direct}}$ , with a consistency correlation of r=0.9684 between the direct and reconstructed ${\mathrm{FOM}}_{S}$ heads (Table 1). Five-fold cross-validation reproduces this pattern (Figure S4; Table S6 lists fold-wise values), so the test split is representative of in-distribution interpolation rather than a one-split accident. Held-out ${\mathrm{FOM}}_{S}$ MAPE remained high in original units—a scale artifact from the eleven-order dynamic range rather than a sign of poor fit.

All baseline models were evaluated with the same fixed 80/10/10 data partition, the same log-transformed targets, and the same V1–V3 OOD validation suite. XGBoost and Random Forest were trained as three independent single-output tree models on $\log_{10}Q_{\mathrm{sc},\mathrm{MACRS}}$ , $\log_{10}C_{\mathrm{sum}}^{-1}$ , and $\log_{10}{\mathrm{FOM}}_{S}$ , whereas the MLP and transformer used the training-fitted scaling described in Section 2 [49,50]. On the fixed in-distribution test split, both tree baselines were strong: XGBoost reached ${\mathrm{FOM}}_{S}$ $R_{\log_{10}}^{2}$ = 0.9868±0.0007 , and Random Forest reached 0.9903±0.0001 , compared with 0.9817 for the transformer. Under out-of-distribution (OOD) evaluation, however, the mean ${\mathrm{FOM}}_{S}$ $R_{\log_{10}}^{2}$ across V1–V3 dropped to 0.2629 for XGBoost and 0.6819 for Random Forest, whereas the transformer retained a mean OOD ${\mathrm{FOM}}_{S}$ $R_{\log_{10}}^{2}$ of 0.9304. When all 43 external points were pooled, the transformer reached ${\mathrm{FOM}}_{S}$ $R_{\log_{10}}^{2}$ = 0.9140 with consistency correlation 0.9660. The transformer was therefore retained for its OOD stability and consistent channel predictions rather than for maximizing accuracy on a random split [51].

**Figure 2:**
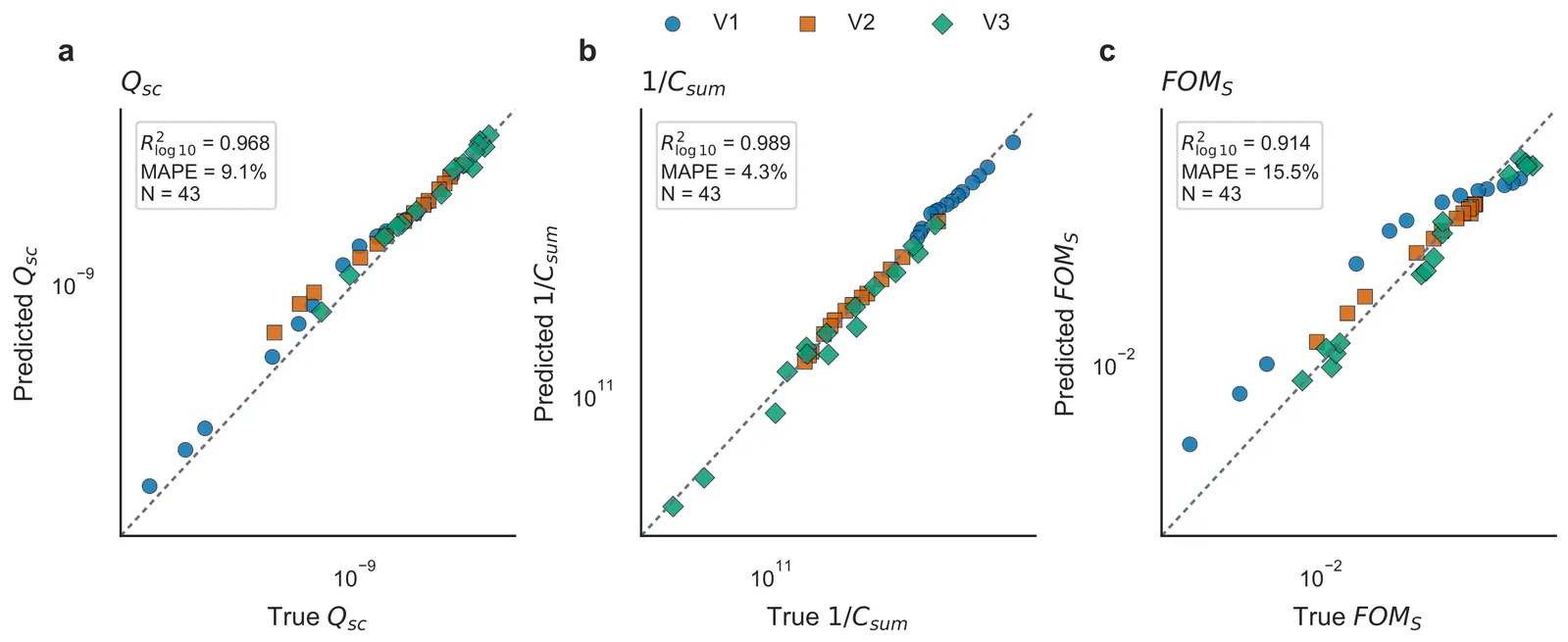
OOD validation on 43 unseen structural–dielectric combinations. (**a**–**c**) compare predictions and ground truth for $Q_{\mathrm{sc},\mathrm{MACRS}}$ , $C_{\mathrm{sum}}^{-1}$ , and ${\mathrm{FOM}}_{S}$ across the three external validation sets V1–V3 on logarithmic axes. The dashed diagonal indicates ideal agreement. The pooled metrics shown in the figure summarize the combined 43-point validation suite, for which the surrogate reaches $R_{\log_{10}}^{2}=0.968$ for $Q_{\mathrm{sc},\mathrm{MACRS}}$ , 0.989 for $C_{\mathrm{sum}}^{-1}$ , and 0.914 for ${\mathrm{FOM}}_{S}$ . The bounded agreement across all three scenarios establishes the validated structural–dielectric domain in which the mechanism and tolerance analyses below are meaningful.

The 43 unseen validation points summarized in Figure 2 provide a bounded check of model performance outside the original training grid. Per-set ${\mathrm{FOM}}_{S}$ $R_{\log_{10}}^{2}$ values are 0.8721 for V1, 0.9538 for V2, and 0.9652 for V3; pooling all 43 points gives an aggregated OOD ${\mathrm{FOM}}_{S}$ $R_{\log_{10}}^{2}$ of 0.9140 with consistency correlation 0.9660 (Table 1). Among the three outputs, $C_{\mathrm{sum}}^{-1}$ is most stable under extrapolation, followed by $Q_{\mathrm{sc},\mathrm{MACRS}}$ ; ${\mathrm{FOM}}_{S}$ shows the widest spread because prediction errors in either channel compound in the reconstructed product. V3 included both seen and unseen *n* values across three new (ε,d/R,h/R) scenarios, and its ${\mathrm{FOM}}_{S}$ $R_{\log_{10}}^{2}$ of 0.9652 confirms that performance does not degrade simply because *n* is new (Figure S5). The more challenging factor is scenario shift—moving into unseen structural and dielectric conditions—rather than unseen electrode-pair counts alone.

### 3.2. Three Distinct Mechanism Regimes Coexist in the Design Space

**Figure 3:**
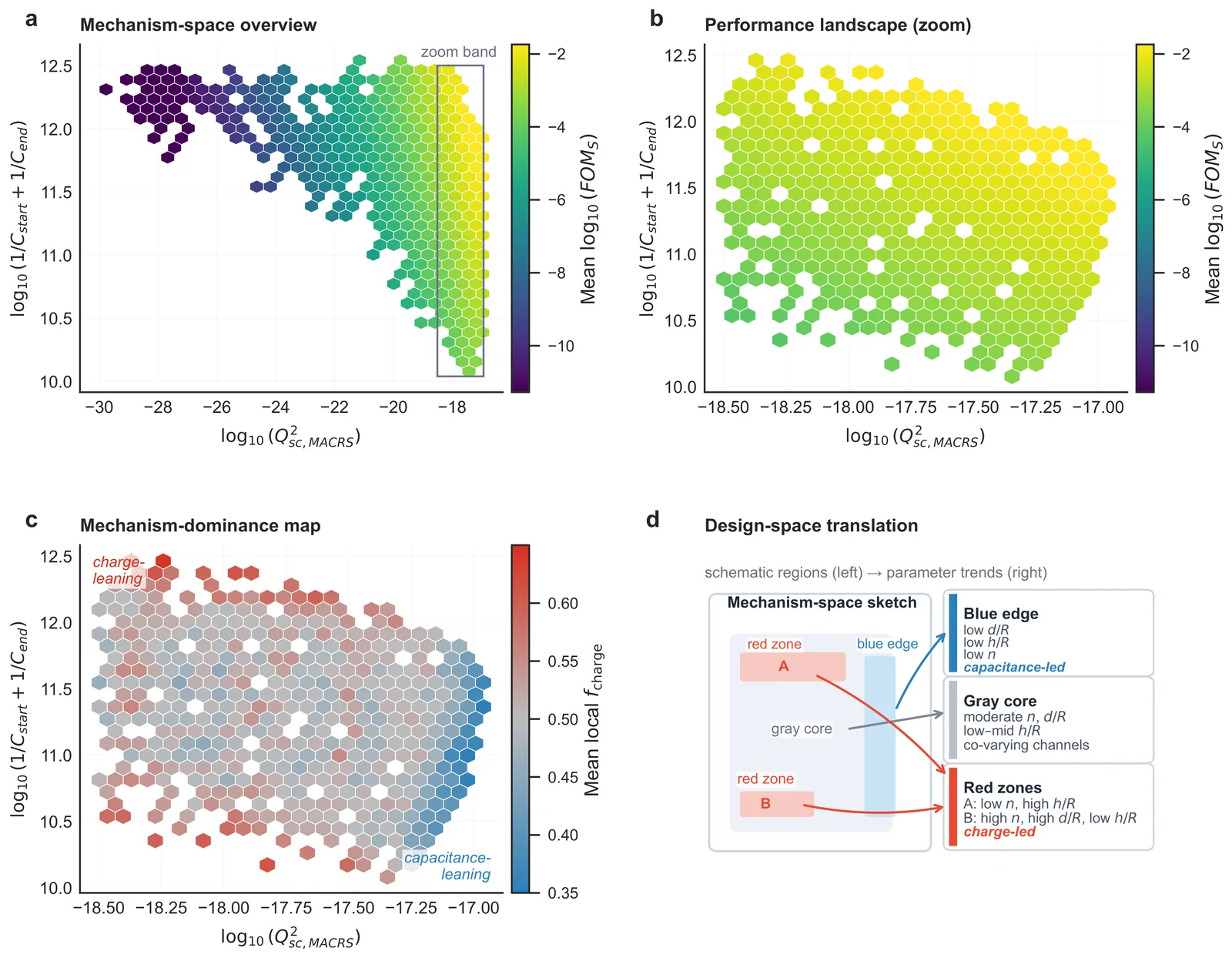
Mechanism-space (charge-transfer × capacitance plane) overview of the disk-TENG design space. (**a**) shows the global distribution of designs in the $\left(\log_{10}Q_{\mathrm{sc},\mathrm{MACRS}}^{2},\log_{10}C_{\mathrm{sum}}^{-1}\right)$ plane colored by mean $\log_{10}\left({\mathrm{FOM}}_{S}\right)$ . (**b**) zooms into the high-performance band that concentrates most of the practically relevant candidates. (**c**) gives the local charge-fraction statistic under the reference classification setting ( k=50 , dominance threshold = 0.62), distinguishing charge-leaning, mixed, and capacitance-leaning regions. (**d**) maps these mechanism-space regions back to representative parameter tendencies in design space, including low- d/R , low- h/R , and different *n* trends.

The dominance landscape in mechanism space (Figure 3) reveals that disk-TENG performance is not governed by a single uniform limiting process. Under the reference setting ( k=50 , dominance threshold = 0.62), 58.6% of the populated region of the mechanism map was classified as charge-dominant, 5.3% as capacitance-dominant, and 36.1% as mixed. These area fractions are setting-specific and should not be read as precise constants; their sensitivity to threshold and neighborhood size is documented in Figure S6. The qualitative conclusion is robust: charge-dominant behavior consistently occupies a far larger fraction of the mechanism map than capacitance-dominant behavior. Over the full parameter sweep ( k=20 to 100, threshold 0.55 to 0.70), the charge-to-capacitance area ratio never drops below 6.2. Sensitivity analyses (Figures S6 and S7) confirm that absolute regime fractions shift continuously with threshold and neighborhood size, yet the qualitative asymmetry between charge and capacitance fractions persists across the entire tested sweep, and grid refinement from 9 to 36 h/R points preserves pairwise label agreement above 0.92.

High-performance designs concentrate within a narrow band of mechanism space rather than spreading uniformly across the full support. Within that band, performance level and mechanism dominance do not coincide: high ${\mathrm{FOM}}_{S}$ values appear across multiple local dominance states, indicating that strong performance can be achieved through different underlying mechanisms.

Figure 3d maps these observations back to the underlying structural parameters. In the high-performance band, three distinct regions are visible: a capacitance-leaning edge at low d/R , low h/R , and low-to-moderate *n*; a mixed core at intermediate parameter combinations; and charge-leaning zones under distinct structural configurations. This confirms that design parameters do not play the same physical role everywhere. Their effect on ${\mathrm{FOM}}_{S}$ depends on which mechanism channel is locally limiting.

After interpolation onto 7776 design points, ${\mathrm{FOM}}_{S,\mathrm{direct}}$ and ${\mathrm{FOM}}_{S,\mathrm{phys}}$ remained highly consistent (Pearson r=0.9988 , Spearman ρ=0.9947 ), confirming that the channel predictions stay coherent when transferred from the discrete simulation grid to the dense landscape.

### 3.3. Design Windows Under Tolerance Screening

**Figure 4:**
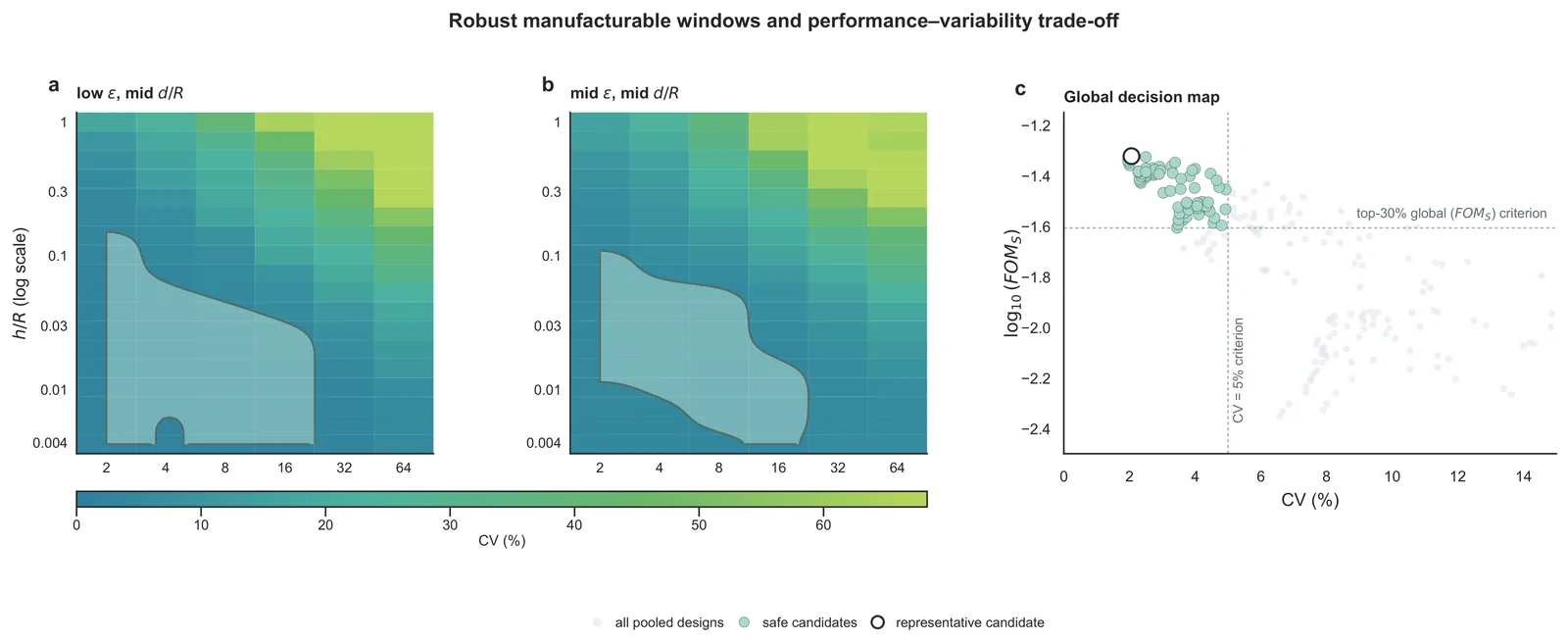
Design screening under symmetric geometric perturbations. (**a**,**b**) show ${\mathrm{FOM}}_{S}$ coefficient of variation (CV) under the applied ±10% perturbation protocol for representative low- and mid-dielectric scenarios, with highlighted windows marking locally favorable robustness zones. (**c**) pools all evaluated candidates into a decision map using CV and $\log_{10}\left({\mathrm{FOM}}_{S}\right)$ . The highlighted “safe candidates” region is defined by the joint criterion CV<5% and top-30% global ${\mathrm{FOM}}_{S}$ , and the highlighted point corresponds to the low- ε , mid- d/R scenario. The safe-candidate region does not overlap with the absolute ${\mathrm{FOM}}_{S}$ peak: the peak frontier sits in a steep-gradient zone where small geometric deviations erode output disproportionately.

Maximizing ${\mathrm{FOM}}_{S}$ and minimizing fabrication sensitivity are not the same objective. The robustness analysis in Figure 4 shows that the highest nominal ${\mathrm{FOM}}_{S}$ and the lowest perturbation sensitivity occupy different regions of the design space, so a screening step that accounts for tolerance is needed before committing any optimum to fabrication.

The ±10% perturbation level was selected as an engineering-scale tolerance proxy rather than as a universal fabrication-error distribution. It provides a controlled way to probe how geometric changes in d/R and h/R propagate through the charge and capacitance channels. Prior disk-TENG studies show that air gap, spacing, and structural configuration materially affect output [8,9], but those studies should be read as support for geometry sensitivity, not as evidence that ±10% is a standard fabrication tolerance.

Under the ±10% symmetric perturbation protocol applied to d/R and h/R , the median ${\mathrm{FOM}}_{S}$ coefficient of variation was 6.76% for (ε=1, d/R=0.125) , 8.16% for (ε=3, d/R=0.125) , 10.71% for (ε=10,d/R=0.125) , and 17.16% for (ε=3,d/R=0.5) . Median worst-case retention remained above 87% in the first three scenarios but dropped to 77.6% in the large-gap case. The minimum retention across all scenarios reached 2.7% at low ε and small d/R . The nominal performance frontier therefore sits in regions with steep local gradients, where small geometric deviations produce disproportionately large output changes.

The pooled decision map (Figure 4c) converts this observation into a practical screening rule. The map plots each candidate as a point in (CV, ${\mathrm{FOM}}_{S}$ ) space, following the Ashby approach of simultaneously displaying two competing objectives. The highlighted “safe candidates” region is defined by the joint criterion CV<5% and top-30% global ${\mathrm{FOM}}_{S}$ . Under this criterion, 62 of 324 pooled candidates entered the high-performance, low-variability set. These candidates cluster in the low-variability, high-performance corner of the decision map and do not coincide with the absolute ${\mathrm{FOM}}_{S}$ peak.

The top-5% ${\mathrm{FOM}}_{S}$ frontier concentrates at small d/R and small h/R —a region dominated by a single channel and therefore highly sensitive to geometric perturbation. The broader top-5% to top-30% performance band shifts toward larger mean d/R and h/R values and lies closer to the mixed regime, where perturbation sensitivity is lower. Across the four representative scenarios, the mixed region occupies 19.4–28.7% of the design maps, and under the present perturbation protocol, that zone offers the most attractive balance between nominal output and perturbation tolerance.

### 3.4. Mechanism Transitions Translate into Conditional Design Rules

**Figure 5:**
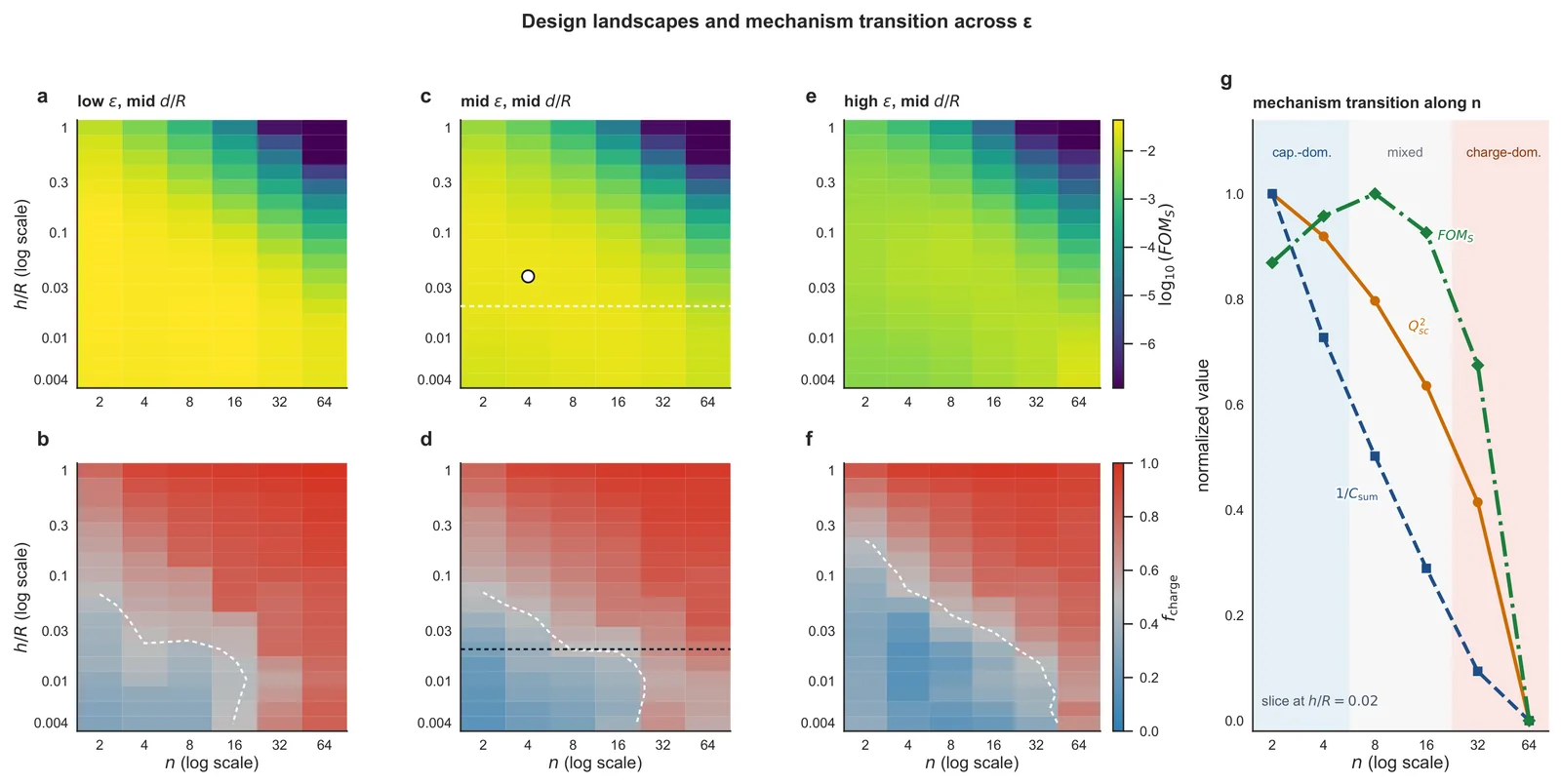
Design landscapes and mechanism transition across dielectric conditions. (**a**,**c**,**e**) show $\log_{10}\left({\mathrm{FOM}}_{S}\right)$ on the (n,h/R) plane for low-, intermediate-, and high-dielectric scenarios at fixed mid-gap conditions, while the paired (**b**,**d**,**f**) show the corresponding local-sensitivity dominance maps under the same reference threshold used for Figure 3. (**g**) follows a representative slice at h/R≈0.02 and compares normalized $Q_{\mathrm{sc},\mathrm{MACRS}}^{2}$ , $C_{\mathrm{sum}}^{-1}$ , and ${\mathrm{FOM}}_{S}$ across *n*. The dashed lines indicate the representative slice used in panel (**g**). The composite ${\mathrm{FOM}}_{S}$ response peaks inside the mechanism transition region rather than at either pure channel extreme, indicating that the most favorable operating point lies where neither channel has yet collapsed to its minimum.

These mechanism transitions translate directly into actionable design rules, summarized in Table 2. Increasing the dielectric constant shifts the dominant mechanism toward capacitance. In the local-sensitivity maps at d/R=0.125 (Figure 5), raising ε from 1 to 10 reduced the charge-dominant fraction from 62.0% to 52.8% and increased the capacitance-dominant fraction from 9.3% to 27.8%; the mixed fraction contracted from 28.7% to 19.4%.

These transitions have a direct physical basis in the ${\mathrm{FOM}}_{S}$ channel decomposition [26,29]. In the Zi et al. framework, the structural figure of merit separates performance into a charge-transfer pathway ( $Q_{\mathrm{sc},\mathrm{MACRS}}$ ) and a capacitance pathway ( $C_{\mathrm{sum}}^{-1}$ ), each depending on geometry. For disk TENGs, increasing ε raises the dielectric-layer capacitance, which reduces $C_{\mathrm{sum}}^{-1}$ . When $Q_{\mathrm{sc},\mathrm{MACRS}}$ variation is already near saturation at a given (n,h/R,d/R) combination, further reduction in $C_{\mathrm{sum}}^{-1}$ begins to control the marginal sensitivity of ${\mathrm{FOM}}_{S}$ ; the mechanism classification therefore shifts toward capacitance-leaning or capacitance-dominant behavior. Conversely, increasing d/R enlarges the air gap relative to the disk radius, reducing effective charge transfer and reinforcing charge dominance [8,10]. In the mixed regime, the local sensitivity is distributed between the charge-transfer and capacitance pathways, so a small geometric perturbation is less likely to drive the design along a single rapidly degrading channel; since $\log{\mathrm{FOM}}_{S}\propto 2\log Q_{\mathrm{sc},\mathrm{MACRS}}+\log C_{\mathrm{sum}}^{-1}$ , balanced partial derivatives with respect to geometry yield lower total sensitivity and more stable retention.

Increasing the air gap has the opposite effect and reinforces charge dominance. At fixed ε=3 , raising d/R from 0.125 to 0.5 increased the charge-dominant fraction from 58.3% to 72.2% and reduced the capacitance-dominant fraction from 16.7% to 8.3%. A larger air gap therefore drives the design toward charge-limited behavior even under moderate dielectric conditions.

At h/R≈0.02 in Figure 5g, both normalized $Q_{\mathrm{sc},\mathrm{MACRS}}^{2}$ and normalized $C_{\mathrm{sum}}^{-1}$ declined monotonically as *n* increased, yet ${\mathrm{FOM}}_{S}$ peaked in the intermediate-*n* range. The best composite performance therefore appears near a transition zone where neither channel has yet fallen to its minimum, rather than at an extreme of either channel alone—a counterintuitive outcome that surrogates predicting only ${\mathrm{FOM}}_{S}$ cannot resolve.

In charge-limited regions, thinner dielectrics and lower sector counts remain effective because charge transfer continues to control the local response. In capacitance-leaning regions, thicker dielectrics and higher *n* values become more viable because dielectric enhancement has already reduced the penalty on the capacitive channel. The dominant optimization channel therefore depends jointly on ε and d/R , so material choice and geometric scoping should be decided together. Mixed regime windows consistently provide the best balance between performance and stability.

## 4. Discussion

### 4.1. Implications for Functional Device Design

Material selection and structural scoping should be decided together rather than in sequence [13,26,28]. Dielectric constant and the air-gap-to-radius ratio jointly determine which mechanism dominates: low ε or large d/R keeps the design charge-limited, while high ε combined with small d/R shifts it toward capacitance-limited behavior. Under charge-limited conditions, reducing h/R and using modest *n* values are the more effective levers. Under capacitance-limited conditions, increasing h/R and raising *n* become viable at a smaller performance cost.

The dielectric-constant axis maps directly onto common TENG triboelectric layers: ε≈2 corresponds to PTFE, ε≈3.5 to Kapton, and ε≈8 –10 to PVDF and its copolymers. Choosing among these materials therefore implicitly selects a starting point in the mechanism landscape, and the conditional rules above translate that material choice into a geometric prescription rather than leaving it for trial and error.

Candidates that maximize nominal ${\mathrm{FOM}}_{S}$ concentrate at small d/R and small h/R , where the performance gradient is steep. Under ±10% geometric perturbation, worst-case retention dropped to as low as 2.7% at the low- ε , small- d/R extreme, and median CV reached 17.2% at large d/R . Mixed-regime windows produced a median CV below 10% across all four representative scenarios. Robustness should therefore enter the design objective rather than serve only as a post hoc filter [47,48,52,53,54,55,56,57].

Table 2 converts these observations into four conditional rules that cover the typical workflow steps: when to prioritize charge enhancement, when capacitance tuning becomes more effective, when to target a mixed-regime window for better stability, and when to step back from the absolute ${\mathrm{FOM}}_{S}$ frontier. Together with the mechanism maps, the rules answer not only which parameters to tune but also where the nominal frontier should not be pushed.

### 4.2. Relationship to Prior Data-Driven TENG Studies

Decomposing ${\mathrm{FOM}}_{S}$ into $Q_{\mathrm{sc},\mathrm{MACRS}}^{2}$ and $C_{\mathrm{sum}}^{-1}$ resolves disk-TENG optimization into spatially heterogeneous mechanisms rather than a single limiting process [29,58]. The present disk-TENG parameterization is closely aligned with the FEM–ML framework of Han et al. [20], where disk-TENG structural variables such as *d*, *h*, *n*, and ε were used for ${\mathrm{FOM}}_{S}$ -oriented structural evaluation. Han et al. established that COMSOL-generated data, surrogate models, and TreeSHAP interpretation can provide useful structural-design insight for disk and spherical TENGs. The present work builds on that disk-TENG foundation by treating the charge-transfer and capacitance terms not only as intermediate quantities for ${\mathrm{FOM}}_{S}$ calculation, but as explicit predictive channels for mechanism classification and tolerance-aware design screening.

The baseline comparison was performed under equivalent data and evaluation conditions. All models used the same fixed 80/10/10 split (seed = 42), the same V1–V3 OOD sets, and the same log-scale metrics. XGBoost and Random Forest were treated as strong tabular baselines and trained as three independent single-output regressors on the same log-transformed targets [49,50]; XGBoost used GridSearchCV on the training partition, and Random Forest used a compact GridSearchCV under the same split. Neural baselines used input and target scaling fitted only on the training split. This setup deliberately separates random-split interpolation from structural–dielectric scenario shift. On the fixed test split, Random Forest reached the highest ${\mathrm{FOM}}_{S}$ $R_{\log_{10}}^{2}$ , followed by XGBoost and the transformer. Under V1–V3 OOD evaluation, however, the transformer retained higher ${\mathrm{FOM}}_{S}$ stability and channel consistency. The selected model is therefore justified by channel coherence and OOD stability, not by claiming universal superiority in random in-distribution accuracy.

Table 3 positions the present workflow relative to the closest data-driven TENG study. The table is not intended as a competitive accuracy comparison, because the disk parameter foundation is related while the modeling objective, output formulation, validation protocol, and design-screening task differ. Instead, it clarifies how the present study further develops the Han et al. FEM–ML–TreeSHAP framework from ${\mathrm{FOM}}_{S}$ -oriented structural evaluation toward channel-resolved mechanism mapping and tolerance-aware candidate selection.

The advance is therefore not a replacement of the earlier FEM–ML framework, but a finer-grained extension of it within the disk-TENG domain. By making $Q_{\mathrm{sc},\mathrm{MACRS}}$ and $C_{\mathrm{sum}}^{-1}$ explicit outputs, the surrogate can report which channel locally limits ${\mathrm{FOM}}_{S}$ and whether a candidate remains stable under the specified perturbation proxy. These outputs support mechanism-aware and tolerance-aware decisions that are not directly available from a single-scalar ${\mathrm{FOM}}_{S}$ prediction alone.

Figure 3 and Figure 5 carry distinct information. Figure 3 shows which channel drives more of the observed ${\mathrm{FOM}}_{S}$ variation across the sampled design population. Figure 5 shows which channel responds more strongly to a parameter adjustment at a specific operating point. A region can be charge-dominant in Figure 3 yet still respond to changes in ε if the capacitance pathway is near a transition. Both maps are needed to select a tuning direction.

The present study is therefore a screening framework grounded in simulation, not a replacement for experimental characterization. Finite-element modeling of triboelectric structures has been established through combined experimental and theoretical studies on segmented disk TENGs [8,9] and sliding-mode geometries [10], and a comprehensive review confirms that simulation now guides mechanism understanding, structural optimization, and equivalent-circuit analysis across TENG modalities [14]. The ${\mathrm{FOM}}_{S}$ formulation itself separates structural and material contributions [26], providing a physically grounded decomposition target. Han et al. recently showed that COMSOL data combined with ML surrogates and interpretable methods can produce actionable structural design insights for TENG systems [20]. The present workflow follows the same paradigm—building a surrogate from simulation data—while adding mechanism mapping by channel and tolerance screening, capabilities that complement rather than replace experimental validation.

### 4.3. Limitations and Boundary Conditions

These conclusions remain bounded by the present dataset and perturbation setup. The workflow rests on COMSOL-derived data without direct experimental confirmation. The perturbation analysis covers only symmetric geometric deviations in d/R and h/R ; it does not account for *n* discreteness, dielectric-constant variability, or asymmetric fabrication bias.

The ±10% perturbation should therefore be read as a tolerance proxy that isolates geometric sensitivity, not as a claim about the actual distribution of fabrication errors in disk TENGs. Real fabrication deviations are typically asymmetric, correlated across dimensions, and coupled to material and environmental variability. A full probabilistic tolerance analysis would need non-symmetric error distributions, correlated d/R / h/R deviations, dielectric-constant variability, and experimental estimates of charge-decay and humidity effects. The present analysis is a first screening step that identifies where such uncertainty is most likely to erode nominal performance.

Direct experimental validation on fabricated disk-TENG prototypes within the identified mixed-regime windows is the immediate next step. A candidate validation protocol would select two to three designs from the “safe candidates” region of Figure 4c and compare measured outputs against surrogate predictions under controlled conditions. Several real-world factors not captured by the present electrostatic simulation are expected to narrow the predicted tolerance windows. Surface charge decay reduces the effective $Q_{\mathrm{sc},\mathrm{MACRS}}$ over operating time, potentially shifting mechanism labels toward capacitance dominance at longer time scales [29,58]. Surface roughness and fabrication defects introduce local inhomogeneities in the electrode–dielectric interface that the smooth-boundary finite-element model does not represent. Environmental humidity modifies triboelectric charging efficiency [5] and may introduce additional variability beyond the geometric perturbations considered here. Rotational frequency and load impedance, omitted from the quasi-static ${\mathrm{FOM}}_{S}$ framework (Equation (1)), will further modulate output under dynamic operating conditions [27]. These factors define the boundary of the present simulation-based screening framework and will need to be quantified in future fabrication trials.

The training grid samples six d/R values and nine h/R values, so the held-out test split and five-fold cross-validation probe interpolation among neighboring conditions, not generalization to new design regions [51]. The 43-point external validation suite provides a more relevant check, but its coverage of the four-dimensional parameter space remains sparse. In low- ${\mathrm{FOM}}_{S}$ regimes, candidate ranking is more meaningful than pointwise absolute predictions.

The dense mechanism landscapes in Figure 3 and Figure 5 are generated by model inference between sampled grid points, not by direct simulation. Figure 3 should therefore be read as a surrogate-based map of likely mechanism organization rather than a directly simulated phase boundary. Sensitivity analyses in Figures S6 and S7 confirm that the qualitative asymmetry between charge and capacitance fractions holds across different neighborhood sizes, classification thresholds, and grid resolutions. The reported rules are specific to disk TENGs within the parameter ranges covered here; transfer to other TENG architectures has not been validated.

The present ${\mathrm{FOM}}_{S}$ framework is quasi-static. Coupling between rotation frequency, load impedance, and the identified mechanism regimes is not captured by Equation (1) and will be addressed in future work alongside the experimental validation step above.

### 4.4. Open-Source Delivery and Reproducibility

Every figure, metric, and design rule in this paper comes from the same trained surrogate under the same configuration, and that same numerical core is exposed through an open-source Streamlit interface [59,60]. A user enters the four design variables (*n*, h/R , d/R , ε ) and the interface returns a ${\mathrm{FOM}}_{S}$ prediction with the individual $Q_{\mathrm{sc},\mathrm{MACRS}}$ and $C_{\mathrm{sum}}^{-1}$ components, the local mechanism label, and a tolerance score that reuses the CV criterion behind Figure 4. All source code is openly available in the GitHub repository, including the processed datasets, model checkpoints, prediction tables, analysis scripts, and interface code. Readers can re-derive the mechanism maps and tolerance screening results, and extend them to new designs within the validated domain [61,62,63,64]. The empirical scope remains defined by the COMSOL dataset, the external OOD suite, and the perturbation protocol used in this study.

## 5. Conclusions

Decomposing ${\mathrm{FOM}}_{S}$ into charge-transfer and capacitance channels reveals that disk-TENG optimization is governed by spatially heterogeneous mechanisms. Charge-dominant, capacitance-dominant, and mixed regimes each occupy distinct parts of the design space, and each calls for a different tuning direction as ε and d/R vary.

Maximizing nominal ${\mathrm{FOM}}_{S}$ and minimizing fabrication sensitivity are not the same objective. The peak- ${\mathrm{FOM}}_{S}$ frontier concentrates at small d/R and h/R , where ±10% geometric perturbations erode worst-case performance to as low as 2.7%. Mixed-regime windows therefore provide the more practical design target when both output level and perturbation tolerance matter.

Validated on 43 unseen structural and dielectric combinations (pooled OOD ${\mathrm{FOM}}_{S}$ $R_{\log_{10}}^{2}=0.914$ ), the surrogate preserves consistent channel predictions where simpler baselines fail. Combined with the mechanism maps and tolerance screening, and exposed through the open design interface, it supplies what single-output workflows cannot: an indication of which mechanism to target in a given region and a quantitative criterion for choosing candidates that tolerate fabrication variation over nominal maxima, before any device is built.

## Figures and Tables

**Table 1:** Compact model capability summary. Values are rounded from the finalized publication source tables. Test and five-fold cross-validation (CV) summarize interpolation fidelity on the structured grid, and “Unseen structural–dielectric combinations” summarizes pooled behavior on the 43-point external validation suite. All $R^{2}$ values are reported on the logarithmic scale because the targets span multiple orders of magnitude.

Validation Dimension	$Q_{\mathrm{sc},\mathrm{MACRS}}$	$C_{\mathrm{sum}}^{-1}$	${\mathrm{FOM}}_{S}$	Consistency *r*
Test	0.9898	0.9966	0.9817	0.9684
5-fold CV	0.986±0.004	0.995±0.003	0.975±0.011	0.952±0.016
Unseen structural–dielectric combinations	0.9680	0.9890	0.9140	0.9660

**Table 2:** Condensed design rules extracted from Figure 3, Figure 4 and Figure 5. Quantitative anchors indicate the parameter ranges in which each rule applies within the present surrogate-supported domain.

Rule	Applicable Condition	Recommended Action and Quantitative Anchor
1	Material selection and initial scoping	Low ε or large d/R keeps the design charge-limited; high ε and small d/R increase capacitance involvement. The charge-dominant fraction drops from 62.0% to 52.8% as ε increases from 1 to 10 at d/R=0.125 .
2	Parameter tuning after identifying the dominant channel	In charge-limited regions, thinner dielectrics and lower *n* remain effective. In capacitance-leaning regions, thicker dielectrics and larger *n* become more viable.
3	Balancing performance and robustness	Mixed regime windows occupy 19.4–28.7% of the representative maps and provide the best balance between output and perturbation tolerance.
4	Exploratory design under tolerance constraints	High- ${\mathrm{FOM}}_{S}$ and high-robustness regions diverge spatially. Target a robust top-30% performance band rather than the absolute ${\mathrm{FOM}}_{S}$ frontier.

**Table 3:** Positioning of the present disk-TENG workflow relative to the Han et al. FEM–ML framework. Entries describe the role of each workflow rather than ranking numerical performance.

Dimension	Han et al. 2025 [20]	This Work
Relationship to disk-TENG parameter space	Established FEM–ML evaluation for disk TENGs using *d*, *h*, *n*, and ε -type structural variables with ${\mathrm{FOM}}_{S}$ -oriented analysis	Uses the same disk-TENG parameter foundation and focuses on deeper mechanism-resolved analysis within the disk domain
Device scope	Disk TENG plus spherical TENG demonstration	Disk TENG only
Main purpose	Structural performance prediction and interpretable feature-interaction analysis	Channel-resolved mechanism classification and tolerance-aware design screening
Use of $Q_{\mathrm{sc},\mathrm{MACRS}}$ and capacitance	Used in FEM-based ${\mathrm{FOM}}_{S}$ calculation	Predicted as explicit output channels: $Q_{\mathrm{sc},\mathrm{MACRS}}$ , $C_{\mathrm{sum}}^{-1}$ , and ${\mathrm{FOM}}_{S}$
Interpretation level	TreeSHAP-based global/local feature importance and interaction features such as nh	Charge–capacitance dominance maps and local limiting-channel identification
Design output	Important parameters, feature interactions, and optimization trends	Mechanism regimes, conditional tuning rules, and robust candidate windows
Robustness analysis	Not the primary focus	Symmetric geometric perturbation proxy with CV and retention screening

## Data Availability

The original data presented in this study are openly available in GitHub at https://github.com/Shenchen-Liu/Mechanism-resolved-design-of-disk-TENGs (accessed on 9 June 2026). Further inquiries can be directed to the corresponding author.

## Supplementary Materials

The following supporting information can be downloaded at: https://www.mdpi.com/article/10.3390/ma19122607/s1.

## Abbreviations

The following abbreviations are used in this manuscript:

TENG	Triboelectric nanogenerator
FOM	Figure of merit
OOD	Out of distribution
CV	Coefficient of variation
COMSOL	COMSOL Multiphysics

## References

[1] Wang Z.L. (2013). Triboelectric nanogenerators as new energy technology for self-powered systems and as active mechanical and chemical sensors. ACS Nano.

[2] Wang Z.L., Chen J., Lin L. (2015). Progress in triboelectric nanogenerators as a new energy technology and self-powered sensors. Energy Environ. Sci..

[3] Cheng T., Shao J., Wang Z.L. (2023). Triboelectric nanogenerators. Nat. Rev. Methods Prim..

[4] Hu J., Iwamoto M., Chen X. (2024). A Review of Contact Electrification at Diversified Interfaces and Related Applications on Triboelectric Nanogenerator. Nano-Micro Lett..

[5] Lacks D.J., Shinbrot T. (2019). Long-standing and unresolved issues in triboelectric charging. Nat. Rev. Chem..

[6] Zhang R. (2025). Triboelectric Intelligence. SmartSys.

[7] Sun Z., He T., Ren Z., Wang C., Liu X., Zhang Z., Zhou J., Guo X., Yang Y., Lee C. (2025). Moving Toward Human-Like Perception and Sensation Systems—From Integrated Intelligent Systems to Decentralized Smart Devices. SmartSys.

[8] Lin L., Wang S., Xie Y., Jing Q., Niu S., Hu Y., Wang Z.L. (2013). Segmentally structured disk triboelectric nanogenerator for harvesting rotational mechanical energy. Nano Lett..

[9] Lin L., Wang S., Niu S., Liu C., Xie Y., Wang Z.L. (2014). Noncontact free-rotating disk triboelectric nanogenerator as a sustainable energy harvester and self-powered mechanical sensor. ACS Appl. Mater. Interfaces.

[10] Niu S., Liu Y., Wang S., Lin L., Zhou Y.S., Hu Y., Wang Z.L. (2013). Theory of Sliding-Mode Triboelectric Nanogenerators. Adv. Mater..

[11] Niu S., Wang S., Liu Y., Zhou Y.S., Lin L., Hu Y., Pradel K.C., Wang Z.L. (2014). A theoretical study of grating structured triboelectric nanogenerators. Energy Environ. Sci..

[12] Tao X., Wang T., Tan L., Xu F., Chen A., Yang Y., Zhang R., Wang X. (2026). High-Performance Constant Current Triboelectric Nanogenerator for Wind Energy Harvesting and Air Purification. SmartSys.

[13] Dai K., Wang X., Niu S., Yi F., Yin Y., Chen L., Zhang Y., You Z. (2017). Simulation and structure optimization of triboelectric nanogenerators considering the effects of parasitic capacitance. Nano Res..

[14] Li W., Guo Y., Wang K., Zhang S., Qiu J., Li J., Suk C.H., Wu C., Zhou X., Zhang Y. (2024). Research advances in triboelectric nanogenerators based on theoretical simulations. Nano Energy.

[15] Yang D., Shang Y., Li Z., Wang K. (2023). Triboelectric Nanogenerator Assisted by Machine Learning. ACS Appl. Electron. Mater..

[16] Zhong X., Gallagher B., Liu S., Kailkhura B., Hiszpanski A., Han T.Y.J. (2022). Explainable machine learning in materials science. npj Comput. Mater..

[17] Butler K.T., Davies D.W., Cartwright H., Isayev O., Walsh A. (2018). Machine learning for molecular and materials science. Nature.

[18] Schmidt J., Marques M.R.G., Botti S., Marques M.A.L. (2019). Recent advances and applications of machine learning in solid-state materials science. npj Comput. Mater..

[19] Himanen L., Geurts A., Foster A.S., Rinke P. (2019). Data-Driven Materials Science: Status, Challenges, and Perspectives. Adv. Sci..

[20] Han C., Jin M., Dong F., Xu P., Jiang X., Cai S.T., Jiang Y., Zhang Y., Fang Y., Niu S. (2025). Interpretable Machine Learning for Evaluating Nanogenerators’ Structural Design. ACS Nano.

[21] Lundberg S.M., Lee S.I. A Unified Approach to Interpreting Model Predictions. Proceedings of the Advances in Neural Information Processing Systems 30.

[22] Lundberg S.M., Erion G., Chen H., DeGrave A., Prutkin J.M., Nair B., Katz R., Himmelfarb J., Bansal N., Lee S.I. (2020). From local explanations to global understanding with explainable AI for trees. Nat. Mach. Intell..

[23] Barredo Arrieta A., Díaz-Rodríguez N., Del Ser J., Bennetot A., Tabik S., Barbado A., García S., Gil-López S., Molina D., Benjamins R. (2020). Explainable Artificial Intelligence (XAI): Concepts, taxonomies, opportunities and challenges toward responsible AI. Inf. Fusion.

[24] Murdoch W.J., Singh C., Kumbier K., Abbasi-Asl R., Yu B. (2019). Definitions, methods, and applications in interpretable machine learning. Proc. Natl. Acad. Sci. USA.

[25] Rudin C. (2019). Stop explaining black box machine learning models for high stakes decisions and use interpretable models instead. Nat. Mach. Intell..

[26] Zi Y., Niu S., Wang J., Wen Z., Tang W., Wang Z.L. (2015). Standards and figure-of-merits for quantifying the performance of triboelectric nanogenerators. Nat. Commun..

[27] Shao J., Jiang T., Tang W., Chen X., Xu L., Wang Z.L. (2018). Structural figure-of-merits of triboelectric nanogenerators at powering loads. Nano Energy.

[28] Peng J., Kang S.D., Snyder G.J. (2017). Optimization principles and the figure of merit for triboelectric generators. Sci. Adv..

[29] Niu S., Wang Z.L. (2015). Theoretical systems of triboelectric nanogenerators. Nano Energy.

[30] Niu S., Liu Y., Zhou Y.S., Wang S., Lin L., Wang Z.L. (2015). Optimization of triboelectric nanogenerator charging systems for efficient energy harvesting and storage. IEEE Trans. Electron Devices.

[31] Niu S., Wang S., Lin L., Liu Y., Zhou Y.S., Hu Y., Wang Z.L. (2013). Theoretical study of contact-mode triboelectric nanogenerators as an effective power source. Energy Environ. Sci..

[32] Niu S., Liu Y., Chen X., Wang S., Zhou Y.S., Lin L., Xie Y., Wang Z.L. (2015). Theory of freestanding triboelectric-layer-based nanogenerators. Nano Energy.

[33] Niu S., Liu Y., Wang S., Lin L., Zhou Y.S., Hu Y., Wang Z.L. (2014). Theoretical Investigation and Structural Optimization of Single-Electrode Triboelectric Nanogenerators. Adv. Funct. Mater..

[34] Niu S., Zhou Y.S., Wang S., Liu Y., Lin L., Bando Y., Wang Z.L. (2014). Simulation method for optimizing the performance of an integrated triboelectric nanogenerator energy harvesting system. Nano Energy.

[35] Shao J., Willatzen M., Jiang T., Tang W., Chen X., Wang J., Wang Z.L. (2019). Quantifying the power output and structural figure-of-merits of triboelectric nanogenerators in a charging system starting from the Maxwell’s displacement current. Nano Energy.

[36] Vaswani A., Shazeer N., Parmar N., Uszkoreit J., Jones L., Gomez A.N., Kaiser L., Polosukhin I. Attention Is All You Need. Proceedings of the Advances in Neural Information Processing Systems 30.

[37] Caruana R. (1997). Multitask Learning. Mach. Learn..

[38] Hwang S., Choi S.K. (2021). Deep learning-based surrogate modeling via physics-informed artificial image (PiAI) for strongly coupled multidisciplinary engineering systems. Knowl.-Based Syst..

[39] Raissi M., Perdikaris P., Karniadakis G.E. (2019). Physics-informed neural networks: A deep learning framework for solving forward and inverse problems involving nonlinear partial differential equations. J. Comput. Phys..

[40] Karniadakis G.E., Kevrekidis I.G., Lu L., Perdikaris P., Wang S., Yang L. (2021). Physics-informed machine learning. Nat. Rev. Phys..

[41] Loshchilov I., Hutter F. Decoupled Weight Decay Regularization. Proceedings of the International Conference on Learning Representations.

[42] Loshchilov I., Hutter F. SGDR: Stochastic Gradient Descent with Warm Restarts. Proceedings of the International Conference on Learning Representations.

[43] Prechelt L. (1998). Early Stopping—But When?. Neural Networks: Tricks of the Trade.

[44] Loftsgaarden D.O., Quesenberry C.P. (1965). A nonparametric estimate of a multivariate density function. Ann. Math. Stat..

[45] Saltelli A. (2002). Sensitivity analysis for importance assessment. Risk Anal..

[46] Hamby D.M. (1994). A review of techniques for parameter sensitivity analysis of environmental models. Environ. Monit. Assess..

[47] Beyer H.G., Sendhoff B. (2007). Robust optimization—A comprehensive survey. Comput. Methods Appl. Mech. Eng..

[48] Goetz S., Schleich B., Wartzack S. (2020). Integration of robust and tolerance design in early stages of the product development process. Res. Eng. Des..

[49] Chen T., Guestrin C. XGBoost: A Scalable Tree Boosting System. Proceedings of the 22nd ACM SIGKDD International Conference on Knowledge Discovery and Data Mining.

[50] Breiman L. (2001). Random Forests. Mach. Learn..

[51] Li K., Rubungo A.N., Lei X., Persaud D., Choudhary K., DeCost B., Dieng A.B., Hattrick-Simpers J. (2025). Probing out-of-distribution generalization in machine learning for materials. Commun. Mater..

[52] Xiu D. (2008). Fast numerical methods for robust optimal design. Eng. Optim..

[53] Kang J.S., Lee T.Y., Lee D.Y. (2012). Robust optimization for engineering design. Eng. Optim..

[54] Zang C., Friswell M.I., Mottershead J.E. (2005). A review of robust optimal design and its application in dynamics. Comput. Struct..

[55] Li W., Gao L., Xiao M. (2020). Multidisciplinary robust design optimization under parameter and model uncertainties. Eng. Optim..

[56] Wang C., Fan H., Qiang X. (2023). A Review of Uncertainty-Based Multidisciplinary Design Optimization Methods Based on Intelligent Strategies. Symmetry.

[57] Lawson J.S., Madrigal J.L. (1994). Robust Design through Optimization Techniques. Qual. Eng..

[58] Shao J., Willatzen M., Wang Z.L. (2020). Theoretical modeling of triboelectric nanogenerators (TENGs). J. Appl. Phys..

[59] Ince D.C., Hatton L., Graham-Cumming J. (2012). The case for open computer programs. Nature.

[60] Wilson G., Aruliah D.A., Brown C.T., Chue Hong N.P., Davis M., Guy R.T., Haddock S.H.D., Huff K.D., Mitchell I.M., Plumbley M.D. (2014). Best Practices for Scientific Computing. PLoS Biol..

[61] Peng R.D. (2011). Reproducible research in computational science. Science.

[62] Sandve G.K., Nekrutenko A., Taylor J., Hovig E. (2013). Ten simple rules for reproducible computational research. PLoS Comput. Biol..

[63] Stodden V., McNutt M., Bailey D.H., Deelman E., Gil Y., Hanson B., Heroux M.A., Ioannidis J.P.A., Taufer M. (2016). Enhancing reproducibility for computational methods. Science.

[64] Wilson G., Bryan J., Cranston K., Kitzes J., Nederbragt L., Teal T.K. (2017). Good enough practices in scientific computing. PLoS Comput. Biol..

