# Supplementary material for: Charge–Capacitance Channel Decomposition Reveals Fabrication-Tolerant Design Windows for Disk Triboelectric Nanogenerators
Source: Materials (Basel). 2026 Jun 17;19(12):2607. doi: 10.3390/ma19122607 (PMC13304420; doi:10.3390/ma19122607)
Supplement: Supplementary file 1 [file materials-19-02607-s001.zip › materials-4340555-supplementary.pdf]

Supporting Information

Charge–Capacitance Channel Decomposition Reveals Fabrication-Tolerant Design Windows for Disk Triboelectric Nanogenerators

Shenchen Liu[1,2], Yangshi Shao[1,2], Xuhong Feng[1,2], Zehui Lin[1,2], Xiaoming Jing[1,2], Everett X. Wang*[1]

[1]*Beijing Institute of Nanoenergy and Nanosystems, Chinese Academy of Sciences, Beijing 101400, China*

[2]*School of Nanoscience and Engineering, University of Chinese Academy of Sciences, Beijing 100049, China*

Overview

The Supporting Information provides supplementary analyses that support the main-text conclusions. Section S1 documents model architecture, training configuration, the per-set composition of the external validation suite, and target distribution statistics with partition-homogeneity tests for reproducibility. Section S2 reports held-out accuracy and channel consistency. Section S3 shows that the model ranking reverses under OOD evaluation. Section S4 verifies that interpolation performance is stable across five folds. Sections S5–S7 confirm that mechanism-map conclusions hold across scenario types, classification thresholds, and grid resolutions. Section S8 documents the open design interface as a delivery layer tied to the same locked numerical core used for the main-text figures.

1 S1. Model architecture, training configuration, and validation set composition

Figure S1 documents the physics-constrained multitask surrogate for reproducibility. The model embeds the four scalar design variables into a shared latent space, processes them with a two-layer transformer encoder using four attention heads, and branches into three output heads for $Q_{\mathrm{sc,MACRS}}$, C_{sum}^{-1}, and $\mathrm{FOM}_{S,\mathrm{direct}}$. A consistency path reconstructs $\mathrm{FOM}_{S,\mathrm{phys}}$ from $Q_{\mathrm{sc,MACRS}}^2 \times C_{\mathrm{sum}}^{-1}$ and ties that reconstruction back to the direct head during training. The later mechanism analysis depends on preserving a coherent relationship between channel predictions and the final scalar target.

The external validation suite consists of three held-out datasets totaling 43 points. Table S2 lists the per-set parameter values; the three sets target progressively harder OOD shifts. V1 fixes the geometric and dielectric variables to grid values while sweeping unseen n values, V2 applies simultaneous shifts in all four variables to a single combined operating point, and V3 spans three structural–dielectric scenarios with both seen and unseen n values per scenario.

Because all three targets span multiple orders of magnitude, distribution statistics are summarized in $\log_{10}$ space (Table S3). $Q_{\mathrm{sc,MACRS}}$ and FOM_S are left-skewed in $\log_{10}$ space (skewness -1.86 and -2.09, respectively), reflecting a long tail of low-output designs at extreme geometric ratios. C_{sum}^{-1} is approximately symmetric (skewness -0.28). The training, validation, and test

*Corresponding author: wangxiaolin@binn.cas.cn; ORCID: 0000-0001-8405-0475 (E.X.W.)

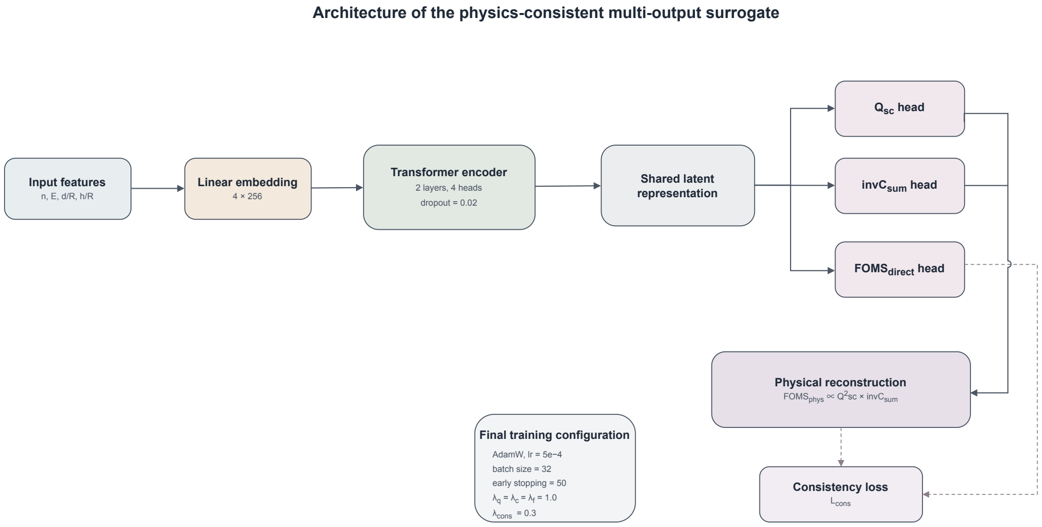

Figure S1: Physics-constrained multi-task surrogate architecture and training pathway. The diagram shows the full path from four scalar design variables to the shared latent representation, the three task heads, the physical reconstruction branch, and the final consistency term, clarifying the model structure and training logic used throughout the analysis.

Table S1: Final hyperparameters and training settings for the reported analysis and deployment workflow.

Hyperparameter	Value
embed_dim	256
nhead	4
num_layers	2
dropout	0.02
lambda_consistency	0.3
lambda_qsc / lambda_invc / lambda_foms	1.0 / 1.0 / 1.0
learning_rate	5×10^{-4}
weight_decay	1×10^{-4}
batch_size	32
max_epochs	300
early_stopping_patience	50
gradient_clip_norm	1.0
lr_scheduler	CosineAnnealingWarmRestarts
T_0 / T_{mult}	50 / 2
seed	42
train_val_test_split	80/10/10
n_total_samples	1944
n_train / n_val / n_test	1555 / 194 / 195

Table S2: Per-set composition of the external validation suite. V1 isolates extrapolation to unseen n values, V2 introduces simultaneous shifts in all four variables, and V3 spans three structural–dielectric scenarios.

Set	ε	d/R	h/R	n values	Samples
V1	1	0.125	0.0625	unseen n values	14
V2	4	0.08	0.05	combined shift	14
V3 (A)	1.5	varied	varied	3, 4, 7, 16, 24	5
V3 (B)	6	varied	varied	3, 4, 7, 16, 24	5
V3 (C)	8	varied	varied	3, 4, 7, 16, 24	5

partitions show comparable target distributions: two-sample Kolmogorov–Smirnov tests yield $p > 0.05$ for all nine partition–target pairs (Table S4), confirming that the fixed-seed 80/10/10 split does not introduce distributional bias.

Table S3: Target distribution statistics in $\log_{10}$ space across the full 1,944-sample dataset.

Statistic	$\log_{10} Q_{\mathrm{sc,MACRS}}$	$\log_{10} C_{\mathrm{sum}}^{-1}$	$\log_{10} \mathrm{FOM}_S$
Min	-15.15	10.08	-12.46
Max	-8.48	12.54	-1.21
Dynamic range (orders)	6.67	2.47	11.25
Median	-9.19	11.58	-2.20
Q1 / Q3	-9.96 / -8.80	11.15 / 11.99	-3.30 / -1.70
IQR	1.16	0.83	1.60
Mean ± std	-9.66 ± 1.30	11.55 ± 0.53	-3.00 ± 2.12
Skewness	-1.86	-0.28	-2.09

Table S4: Kolmogorov–Smirnov tests between data partitions in $\log_{10}$ space. All p-values exceed 0.05, indicating no systematic distributional shift among the training (1,555), validation (194), and test (195) splits.

Partition pair	$\log_{10} Q_{\mathrm{sc,MACRS}}$	$\log_{10} C_{\mathrm{sum}}^{-1}$	$\log_{10} \mathrm{FOM}_S$
Train vs. Val	$D = 0.086$, $p = 0.15$	$D = 0.050$, $p = 0.76$	$D = 0.098$, $p = 0.068$
Train vs. Test	$D = 0.068$, $p = 0.39$	$D = 0.046$, $p = 0.85$	$D = 0.060$, $p = 0.54$
Val vs. Test	$D = 0.099$, $p = 0.27$	$D = 0.070$, $p = 0.69$	$D = 0.099$, $p = 0.28$

2 S2. Held-out prediction remains accurate on the log scale and internally self-consistent

Figure S2 and Table S5 provide the main-text fidelity defense on the structured grid. The key result is not only that $\mathrm{FOM}_{S,\mathrm{direct}}$ tracks the held-out targets well, but that $\mathrm{FOM}_{S,\mathrm{direct}}$ and $\mathrm{FOM}_{S,\mathrm{phys}}$ remain mutually coherent. On the held-out test split, $Q_{\mathrm{sc,MACRS}}$, C_{sum}^{-1}, $\mathrm{FOM}_{S,\mathrm{direct}}$, and $\mathrm{FOM}_{S,\mathrm{phys}}$ reached $R^2_{\log_{10}}$ values of 0.9898, 0.9966, 0.9817, and 0.9841, respectively. The corresponding $\mathrm{MAE}_{\log 10}$ values were 0.0763, 0.0231, 0.1434, and 0.1526.

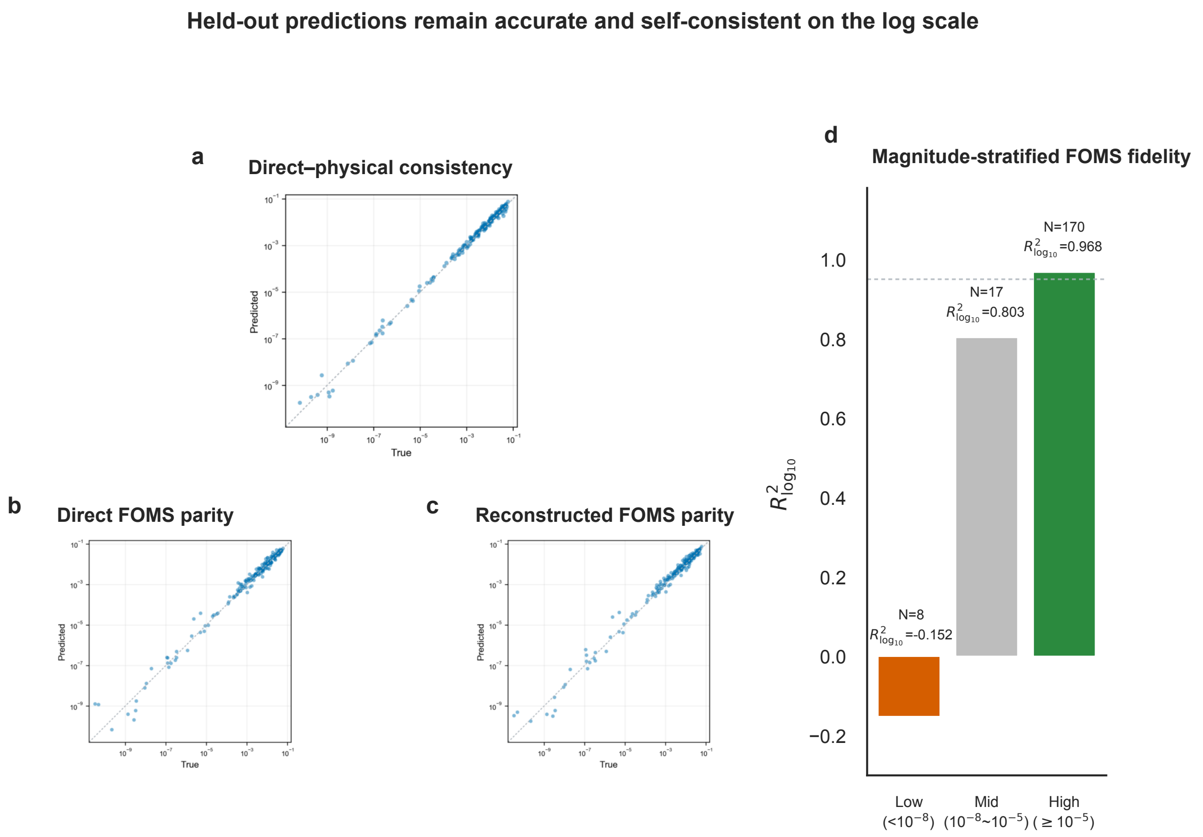

Figure S2: Held-out prediction remains accurate on the log scale and internally self-consistent. The direct-versus-physical panel shows that the two FOM_S pathways remain aligned, the parity panels show that both direct and reconstructed FOM_S preserve the correct logarithmic trend on the held-out split, and the magnitude-band panel shows how fidelity changes across FOM_S scales across the structured grid.

Table S5: Per-head metrics across test, cross-validation, and OOD validation.

Head	Test $R^2_{\log_{10}}$	CV mean	CV std	V1	V2	V3	Test $\mathrm{MAE}_{\log 10}$
$Q_{\mathrm{sc,MACRS}}$	0.9898	0.9863	0.0041	0.9652	0.9091	0.9802	0.0763
C_{sum}^{-1}	0.9966	0.9948	0.0026	0.9726	0.9908	0.9752	0.0231
$\mathrm{FOM}_{S,\mathrm{direct}}$	0.9817	0.9754	0.0108	0.8720	0.9538	0.9653	0.1434
$\mathrm{FOM}_{S,\mathrm{phys}}$	0.9841	0.9798	0.0063	0.8962	0.5518	0.9609	0.1526

3 S3. In-distribution competitiveness does not guarantee OOD generalization

Figure S3 is the model-selection logic figure. On the in-distribution test split, Random Forest achieved the highest FOM_S $R^2_{\log_{10}}$ (0.9903 ± 0.0001), followed by XGBoost (0.9868 ± 0.0007) and the transformer (0.9817). The OOD comparison reverses this ranking. Average OOD FOM_S $R^2_{\log_{10}}$ falls to 0.6819 for Random Forest, 0.2629 for XGBoost, 0.3148 for the multilayer perceptron (MLP) without consistency, and 0.2594 for the MLP with consistency, whereas the retained transformer reaches 0.9304. The strongest contrast appears in V2, where all four baselines degrade sharply in FOM_S $R^2_{\log_{10}}$, while the retained transformer remains at 0.9538 and preserves consistency Pearson $r = 0.9665$.

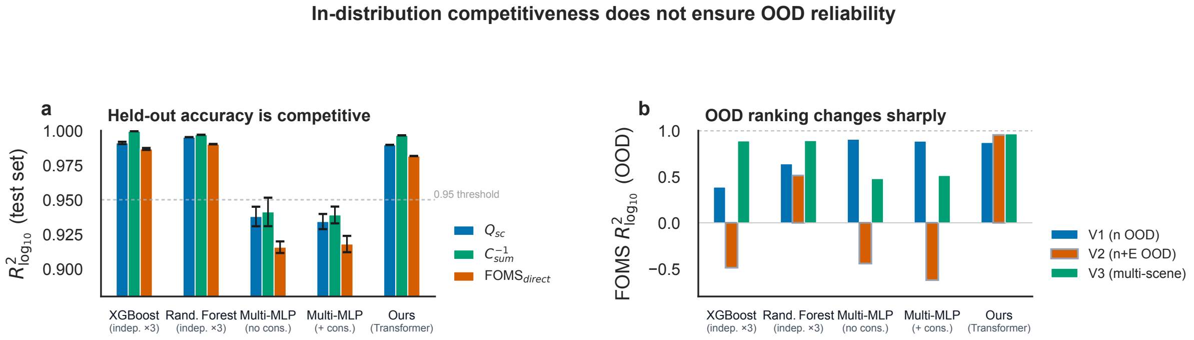

Figure S3: In-distribution competitiveness does not guarantee OOD generalization. The left panel provides the in-distribution context in which strong baselines (including Random Forest) remain competitive, whereas the right panel shows that model ranking changes substantially once structural–dielectric conditions move off distribution.

4 S4. Cross-validation confirms stable interpolation performance and limited overfitting

Figure S4 and Table S6 strengthen the internal-validity argument by showing that held-out performance is not a one-split accident. Across five folds, validation $R^2_{\log_{10}}$ remained high for all four outputs. For $Q_{\mathrm{sc,MACRS}}$, the foldwise values ranged from 0.9781 to 0.9890; for C^{-1}_{sum}, from 0.9897 to 0.9968; for $\mathrm{FOM}_{S,\mathrm{direct}}$, from 0.9562 to 0.9847; and for $\mathrm{FOM}_{S,\mathrm{phys}}$, from 0.9672 to 0.9840. Direct-versus-physical consistency Pearson r remained between 0.9326 and 0.9764 across folds.

Table S6: Fold-wise cross-validation details.

Fold	n_{val}	$Q_{\mathrm{sc,MACRS}}$	C^{-1}_{sum}	$\mathrm{FOM}_{S,\mathrm{direct}}$	$\mathrm{FOM}_{S,\mathrm{phys}}$	Consistency Pearson	Consistency Spearman
1	389	0.9890	0.9962	0.9837	0.9840	0.9584	0.9861
2	389	0.9781	0.9968	0.9562	0.9672	0.9326	0.9689
3	389	0.9873	0.9897	0.9709	0.9820	0.9575	0.9796
4	389	0.9886	0.9958	0.9816	0.9833	0.9345	0.9788
5	388	0.9884	0.9954	0.9847	0.9824	0.9764	0.9953

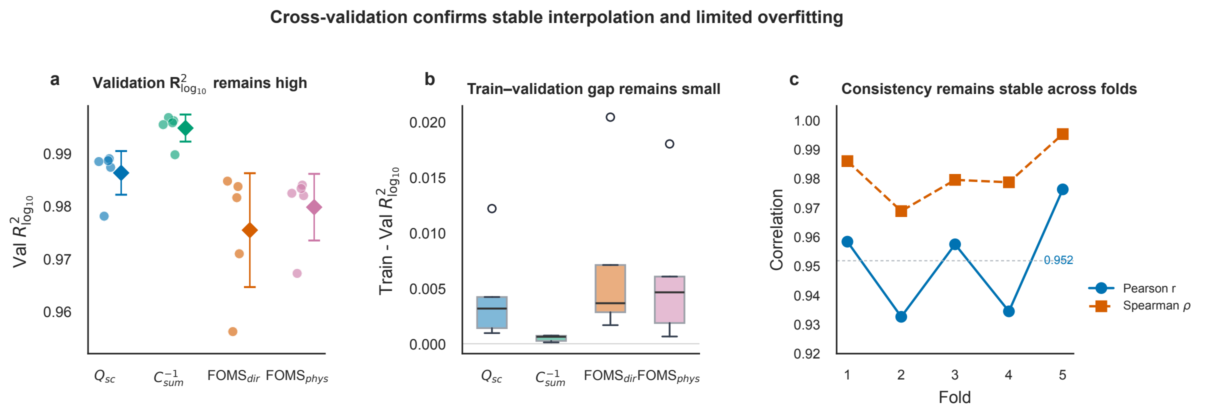

Figure S4: Cross-validation confirms stable interpolation performance and limited overfitting. Fold-wise validation behavior, train-versus-validation separation, and consistency correlations remain stable across repeated retraining on the structured grid.

5 S5. OOD degradation is driven more by scenario shifts than by n extrapolation alone

Figure S5 decomposes the external validation behavior. Within V3, the pooled split between seen and unseen n values shows only a minor difference. For the ID-n subset, FOM$_S$ $R^2_{\log_{10}}$ is 0.9632 with $\mathrm{MAE}_{\log 10} = 0.0443$; for the OOD-$n$ subset, FOM$_S$ $R^2_{\log_{10}}$ is 0.9666 with $\mathrm{MAE}_{\log 10} =$ 0.0426. By contrast, the three five-point scenario slices produce FOM$_{S,\mathrm{direct}}$ MAE$_{\log 10}$ values of 0.0653, 0.0430, and 0.0215 for scenarios A, B, and C, respectively. This pattern supports the main interpretation that scenario shift matters more than unseen n alone.

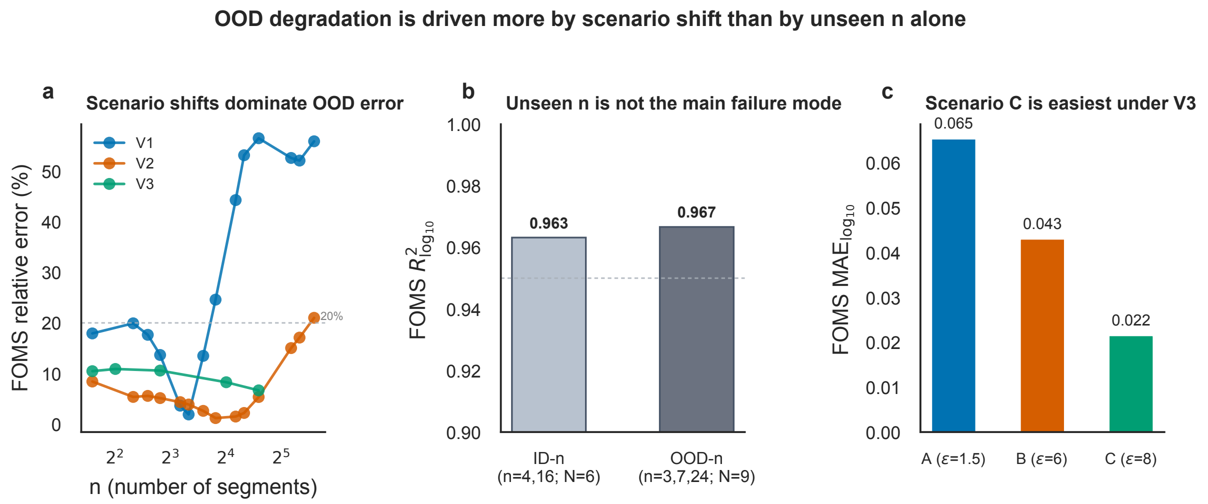

Figure S5: OOD degradation is driven more by scenario shifts than by n extrapolation alone. The panels separate ID-n and OOD-n behavior within V3 and contrast that split with scenario-level error structure across the three V3 subconditions. The close ID-n/OOD-n behavior and the larger scenario-to-scenario spread show that compound structural–dielectric shift is the more important OOD boundary.

6 S6. Regime asymmetry remains robust across threshold and neighborhood choices

Figure S6 addresses the threshold-sensitivity concern directly. Across $k = 20$ to 100 and dominance thresholds from 0.55 to 0.70, the charge-dominant fraction spans 24.60% to 76.85%, the capacitance-dominant fraction spans 2.45% to 9.00%, and the mixed fraction spans 16.50% to 72.25%. The charge-to-capacitance area ratio never falls below 6.23 and reaches as high as 20.00. Under the reference setting of $k = 50$ and threshold 0.62, the fractions are 58.60% charge-dominant, 5.30% capacitance-dominant, and 36.10% mixed.

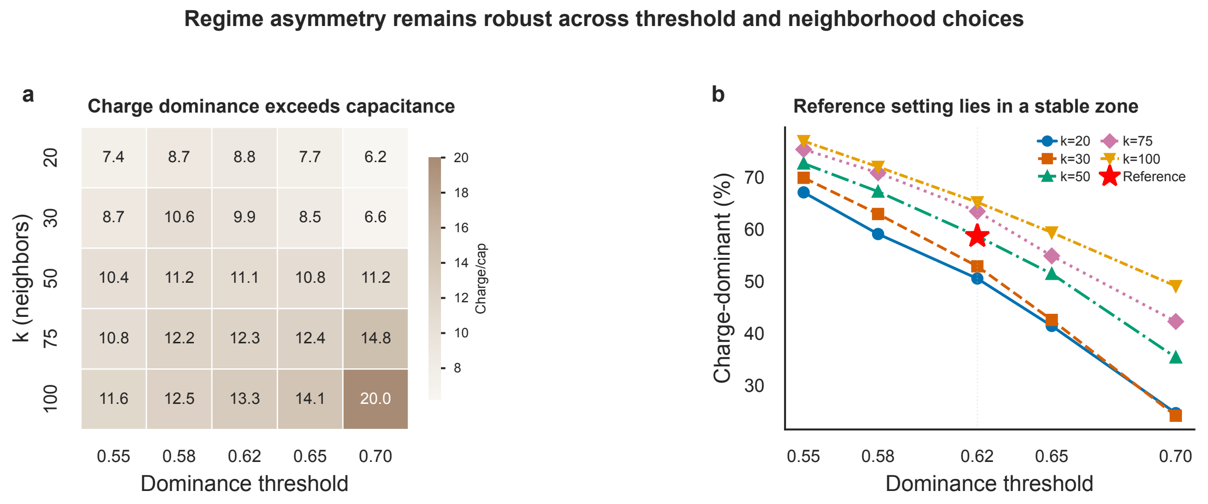

Figure S6: Regime asymmetry remains robust across threshold and neighborhood choices. Absolute regime fractions move continuously as the neighborhood size and dominance threshold are varied, but the qualitative inequality charge-dominant > capacitance-dominant is preserved across the entire tested sweep. The reference setting lies within this stable region.

7 S7. Grid refinement preserves boundary structure and label agreement

Figure S7 addresses the complementary concern that the regime map might be a coarse-grid artifact. When the design-space map is recomputed with the number of h/R grid points set to 9, 18, and 36, the charge-dominant fraction changes only from 59.26% to 58.33%, the capacitance-dominant fraction from 16.67% to 15.74%, and the mixed fraction from 24.07% to 25.93%. Pairwise label agreement is 0.935 between the 9-point and 18-point maps, 0.926 between the 9-point and 36-point maps, and 0.972 between the 18-point and 36-point maps. Mean intersection over union (IoU) rises from 0.825 for the coarsest-to-finest comparison to 0.937 for the two finer grids.

8 S8. Representative views of the open design interface

Figure S8 documents the open design interface used as the delivery layer for the reported workflow. The app exposes single-point prediction, design-space exploration, and tolerance-aware candidate-window screening while reusing the locked multitask surrogate, mechanism metrics, and robustness-screening logic used for the main-text figures. It is released with the associated study resources and is intended for bounded exploration within the validated structural–dielectric domain rather than as an independent source of new scientific results.

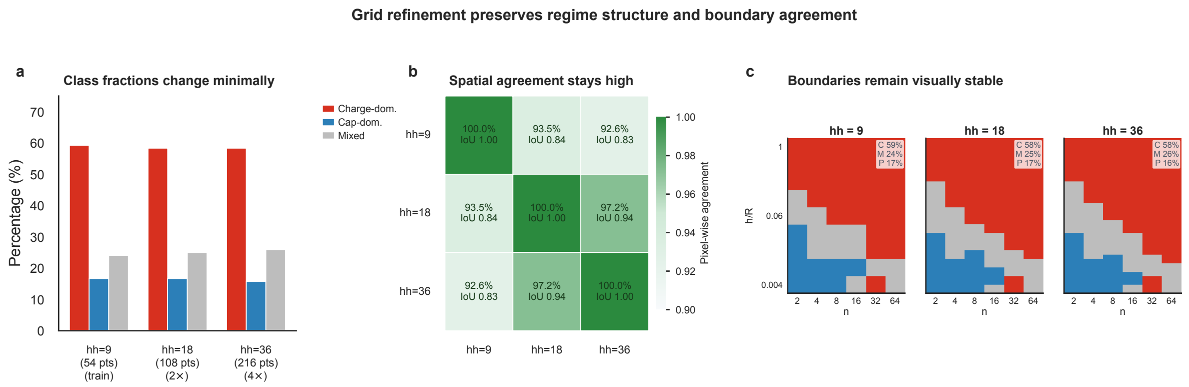

Figure S7: Grid refinement preserves boundary structure and label agreement. Aggregate class fractions remain stable as the map is refined from 9 to 18 to 36 h/R points, and the registered label maps retain high agreement and mean IoU. Refinement changes the boundary smoothly without qualitative map reconstruction.

Supporting note. Consistency-loss ablation

Table S7 records the consistency-loss ablation as a methodological note. On the test set, removing the consistency term changes $Q_{\mathrm{sc,MACRS}}$, C_{sum}^{-1}, and $\mathrm{FOM}_{S,\mathrm{direct}}$ $R^2_{\log_{10}}$ only slightly, but the direct-versus-physical consistency Pearson correlation drops from 0.9684 to 0.9396 ± 0.0247. This is the clearest evidence that the consistency term acts primarily as a structural safeguard.

Table S7: Test-set consistency-loss ablation summary.

Metric	T1 with consistency	T5 no consistency	Δ
$Q_{\mathrm{sc,MACRS}}$ $R^2_{\log_{10}}$	0.9898	0.9835 ± 0.0014	-0.0063
C_{sum}^{-1} $R^2_{\log_{10}}$	0.9966	0.9942 ± 0.0012	-0.0025
$\mathrm{FOM}_{S,\mathrm{direct}}$ $R^2_{\log_{10}}$	0.9817	0.9805 ± 0.0040	-0.0011
Consistency Pearson	0.9684	0.9396 ± 0.0247	-0.0288

Table S8: OOD consistency-loss ablation summary.

Validation set and metric	T1 with consistency	T5 no consistency	Δ
V1 FOM_S $R^2_{\log_{10}}$	0.8720	0.9564	+0.0844
V1 Consistency Pearson	0.9770	0.9700	-0.0070
V2 FOM_S $R^2_{\log_{10}}$	0.9538	0.9489	-0.0049
V2 Consistency Pearson	0.9665	0.9890	+0.0225
V3 FOM_S $R^2_{\log_{10}}$	0.9653	0.9586	-0.0067
V3 Consistency Pearson	0.9886	0.9900	+0.0014

SI conclusion

Taken together, these analyses establish four points. First, the surrogate is reproducible and physically structured. Second, its held-out fidelity on the sampled grid is sufficient to support landscape-oriented analysis. Third, the model was selected over simpler baselines because of its OOD channel coherence, not its in-distribution test score. Fourth, the qualitative mechanism-

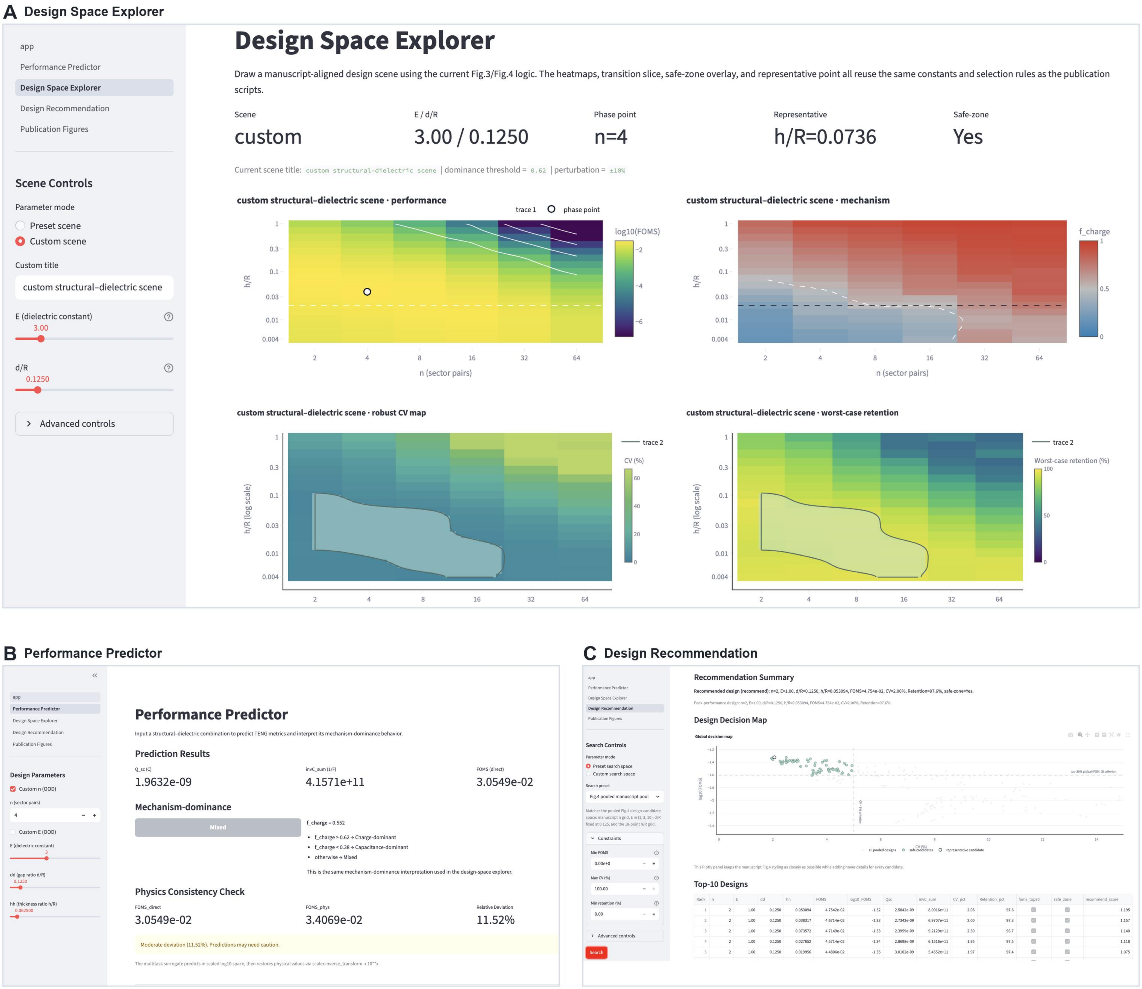

Figure S8: Representative views of the open design interface. The tool reuses the locked multitask surrogate, mechanism metrics, and robustness-screening logic used for the main-text figures. Shown are representative panels for single-point prediction, design-space exploration, and tolerance-aware candidate-window screening within the validated structural–dielectric domain. The interface is provided as a delivery layer for the reported workflow rather than as an independent source of new results.

map conclusions—charge asymmetry, regime boundaries, and robustness windows—are stable across threshold, neighborhood, and grid-refinement choices. The open design interface exposes this same locked numerical core for single-point prediction, design-space exploration, and tolerance-aware candidate screening.
